# Deactivation of the GATA Transcription Factor ELT-2 Is a Major Driver of Normal Aging in *C*. *elegans*

**DOI:** 10.1371/journal.pgen.1005956

**Published:** 2016-04-12

**Authors:** Frederick G. Mann, Eric L. Van Nostrand, Ari E. Friedland, Xiao Liu, Stuart K. Kim

**Affiliations:** 1 Departments of Genetics and Developmental Biology, Stanford University Medical Center, Stanford, California, United States of America; 2 Department of Cellular and Molecular Medicine, Institute of Genomic Medicine, University of California, San Diego, La Jolla, California, United States of America; 3 Editas Medicine, Cambridge, Massachusetts, United States of America; 4 School of Life Sciences, Tsinghua University, Beijing, China; Princeton, UNITED STATES

## Abstract

To understand the molecular processes underlying aging, we screened modENCODE ChIP-seq data to identify transcription factors that bind to age-regulated genes in *C*. *elegans*. The most significant hit was the GATA transcription factor encoded by *elt-2*, which is responsible for inducing expression of intestinal genes during embryogenesis. Expression of ELT-2 decreases during aging, beginning in middle age. We identified genes regulated by ELT-2 in the intestine during embryogenesis, and then showed that these developmental genes markedly decrease in expression as worms grow old. Overexpression of *elt-2* extends lifespan and slows the rate of gene expression changes that occur during normal aging. Thus, our results identify the developmental regulator ELT-2 as a major driver of normal aging in *C*. *elegans*.

## Author Summary

A central question in aging is to uncover the molecular drivers of the normal aging process. Using the roundworm *C*. *elegans* as a model organism, we found that the ELT-2 GATA transcription factor regulates many gene expression changes that occur during aging. During development, *elt-2* functions as a key regulator of intestinal development. During aging, the levels of ELT-2 transcription factor decline, which causes expression of its target genes to decline and this limits lifespan. Reduction of *elt-2* GATA activity in young worms induces gene expression changes resembling those that normally occur in old age. Overexpression of *elt-2* slows down the rate of aging, and extends lifespan by about 25%. This work finds that worm lifespan is limited by the declining expression of a key developmental regulator.

## Introduction

Identifying the factors that govern the rate of aging could illuminate a way to reduce the incidence of many diseases at once. Therefore, it is critical to understand the molecular events that drive the transition from young to old. Yet, the underlying molecular mechanisms that drive the normal process of aging are poorly understood. The nematode worm *Caenorhabditis elegans* is an excellent model to study the normal aging process as it has a lifespan of approximately two weeks and shows signs of aging on many levels. Old worms move slowly and have less pharyngeal pumping [1]. At the tissue level in old age, the intestine loses microvilli and some of its nuclei [2]. The muscles show fragmented fibers indicative of sarcopenia [3]. At the sub-cellular level, old worms accumulate lipofuscin in the intestine, as well as lipids and yolk proteins throughout the body [1,4].

At the level of RNA changes, high-throughput technologies enable thousands of molecules to be profiled in parallel. Gene expression studies have identified over a thousand genes that show expression differences between young and old worms, referred to as the aging transcriptome [5–7]. The age-regulated genes tend to be expressed in the intestine, and have promoters that contain bindings sites for GATA transcription factors [5].

Several upstream regulators of the aging transcriptome have been identified and shown to drive of aging process. During aging, changes in the expression of transcriptional regulators such as ELT-3, ETS-4, UNC-62A, and PQM-1 cause changes in the expression of hundreds of their direct target genes, and modulate lifespan [5,8–10]. MicroRNAs also change expression during aging, thereby altering regulation of downstream targets and acting to both promote and antagonize longevity [11].

ChIP-seq data produced by the modENCODE Consortium has opened up new ways to search for regulators of the normal aging transcriptome in an unbiased manner [10,12,13]. One can screen the set of transcription factor binding sets generated by modENCODE to identify transcription factors that bind to age-regulated genes, thereby generating a candidate list of upstream drivers of gene expression changes in old age [10,13]. This is a powerful strategy because it offers a quantitative and objective way to screen for regulators with the largest impact on the aging transcriptome.

Here, we identify *elt-2*, which encodes a GATA transcription factor homologous to human GATA4, as a direct regulator of the aging transcriptome. *elt-2* is expressed exclusively in the intestine and plays a key role in inducing intestinal gene expression during embryonic development [14]. In adults, reduction of *elt-2* activity by RNAi shortens the lifespan extension caused by mutations in *daf-2*, *eat-2*, and *isp-1*; *ctb-1*, and also increases the toxicity of the pathogenic bacterium *Pseudomonas aeruginosa* [5,15–17].

To investigate the role of *elt-2* during the normal aging process, we first examined the expression of *elt*-*2* over time, and found that it decreases in old age. For the genes regulated by ELT-2 GATA, we found a striking pattern of transcriptional induction during early development followed by reduction during aging. Overexpression of *elt-2* extends lifespan and reduces the magnitude of these age-related changes in gene expression. This transcriptional effect of *elt-2* overexpression is seen not only in the intestine where it is expressed, but also in the muscle, hypodermis and neuronal tissues, indicating that the intestine communicates to other organs to slow down the aging process. These results show that a major aspect of the normal aging process in *C*. *elegans* involves loss of developmental control of the intestine.

## Results

**Fig 1:**
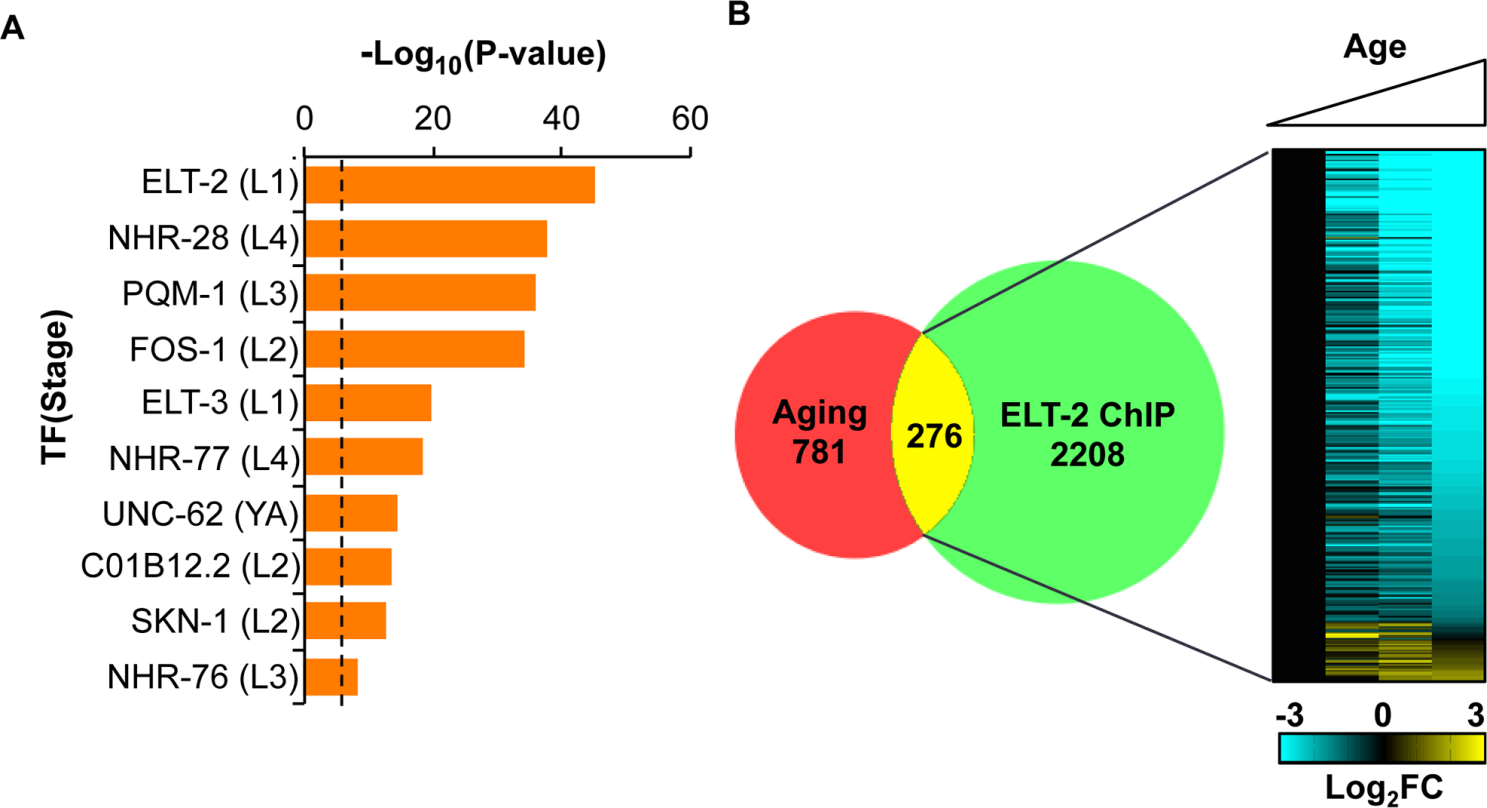
Screen for direct regulators of the aging transcriptome with modENCODE ChIP-seq data. (A) Transcription factors with significant overlap between the aging transcriptome and modENCODE ChIP-seq datasets. 99 ChIP-seq datasets for 58 transcription factors were used. Y-axis denotes Chi-square p-values for overlap. Dotted line represents a Bonfrerroni-corrected significance threshold of p = .05/99 = p<10^−4^. The stage used for the ChIP-seq analysis is denoted in parentheses. (B) Significant overlap between the set of age-regulated genes and low-complexity targets of ELT-2 is shown [5]. Numbers indicate number of genes for each part of the Venn diagram. The heatmap depicts the level of expression for the 276 genes bound by ELT-2 during aging. Columns indicate level of expression at Days 2, 5, 8, and 11 of adulthood at 25°C. Data are presented as Log_2_ fold changes relative to Day 2 for each gene.

To identify transcription factors that are bound to age-regulated genes, we examined 99 ChIP-seq datasets for 58 transcription factors generated by the modENCODE consortium. We screened for datasets in which the set of genes bound by the transcription factor shows a strong overlap with genes that are age-regulated [13]. Rather than screen all of the sites bound by a transcription factor, we narrowed the search by using sites that have a low complexity score. The complexity score indicates the number of transcription factors that are bound to the same site; low complexity scores indicate that the site is bound specifically by that transcription factor (and a few others) and high complexity scores indicate that the site is bound by many transcription factors. Previous work has demonstrated that sites with a low complexity score are more likely to identify genes regulated by a transcription factor than genes with high complexity scores [9,13]. We used sites with a complexity score less than 8 and asked which transcription factor is most enriched for binding age-regulated genes. This screen identified ten transcription factors enriched for binding age-regulated genes using 1.5-fold enrichment and p< 10^−5^ as a cutoff (Fig 1A). Six of the ten factors have previously been shown to extend lifespan in genetic loss- or gain-of-function experiments: (*elt-2*, *nhr-28*, *elt-3*, *unc-62*, *skn-1*, *nhr-76*) [5,9,13,18,19].

The most significant hit on the list is *elt-2*, which encodes one of the fourteen GATA transcription factor genes in the *C*. *elegans* genome. The modENCODE ChIP–seq experiment identified a total of 2484 low-complexity targets bound by ELT-2 in the L1 larval stage (S1 Table). The low complexity regions bound by ELT-2 are strongly enriched for genes that show age-regulation; specifically, 276 of the 2484 low complexity targets overlap the set of 1057 age-regulated genes from Budovskaya *et al*., which is 2.1-fold more than would be expected by chance (Chi-square p < 10^−45^)(Fig 1B, S2 Table).

Previous work has shown that the DNA regions upstream of the age-regulated genes are enriched for the consensus motif bound by GATA transcription factors [5]. *elt-2* shows the strongest enrichment for binding age-regulated genes of the three GATA transcription factor genes tested by modENCODE. In addition, the age-regulated genes are highly-enriched for genes expressed in the intestine [5]. *elt-2* is expressed exclusively in the intestine [14]. Finally, *elt-2* is an important regulator of intestinal differentiation and function. Null mutants for *elt-2* form non-functional intestines, cannot swallow food, and starve shortly after hatching [14]. Ectopic expression of *elt-2* during embryogenesis arrests development and causes nearly all cells to express the intestinal fate [14]. A change in *elt-2* activity during normal aging could disrupt the transcription of hundreds of genes within the intestine; understanding this process might lead to new and different insights about developmental mechanisms that regulate aging.

### Declining expression of ELT-2 during aging limits lifespan

**Fig 2:**
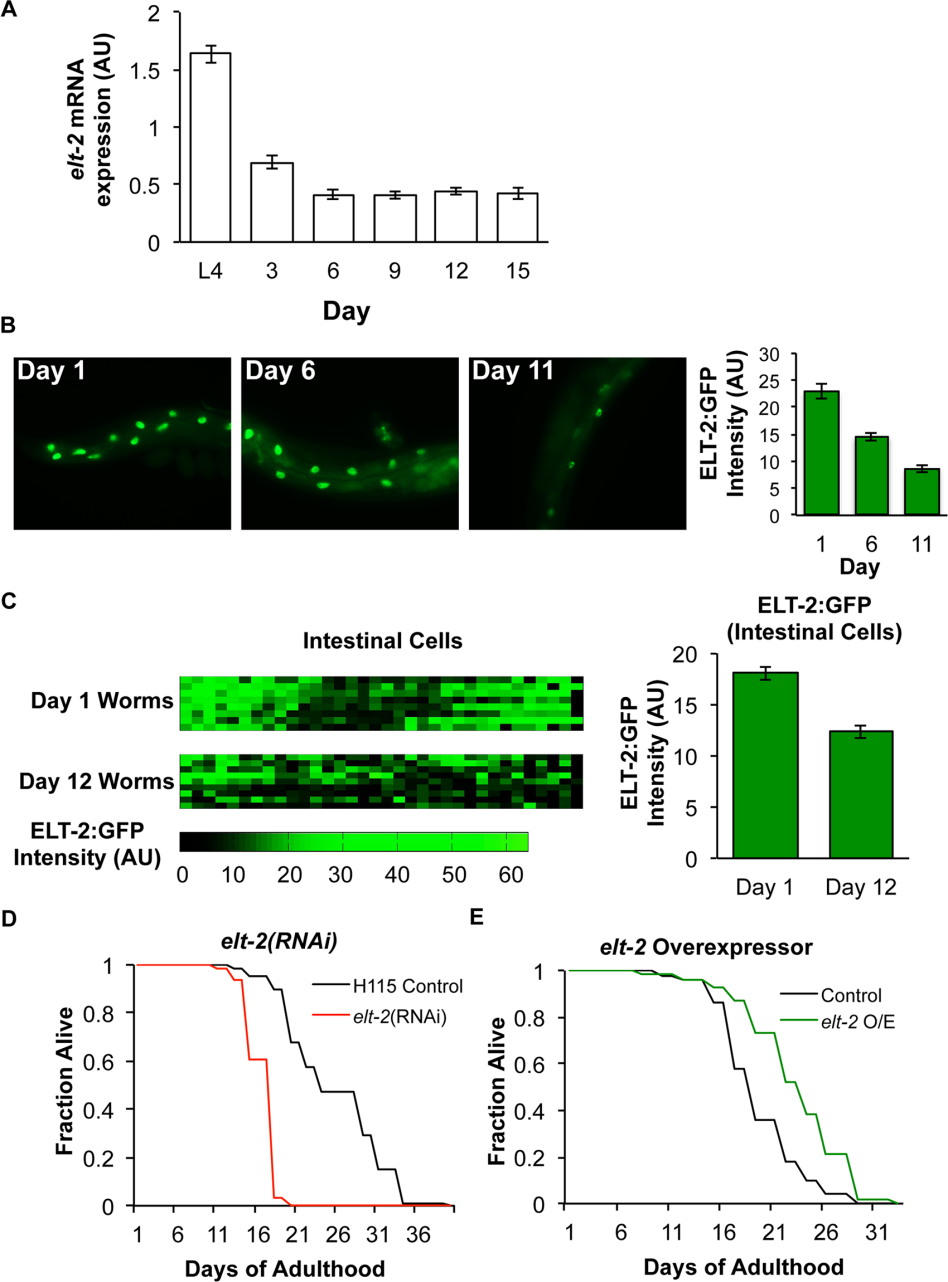
Declining ELT-2 expression during aging limits lifespan. (A) Abundance of *elt-2* mRNA during aging, measured by qRT-PCR. Error bars indicate SEM from three biological replicates. *elt-2* levels decline from L4 to Day 3, and from Day 3 to Day 6 (p < .05, Student’s t-test). (B) Representative images of ELT-2:GFP animals during aging (Days 1, 6 and 11 of adulthood). Bar graph shows quantitation of average GFP intensity per animal from a population of at least 20 animals for each time point. Error bars indicate SEM. ELT-2:GFP levels decline from Day 1 to Day 6 and from Day 6 to Day 11 (p < .05, Student’s t-test). (C) The heatmaps depict ELT-2:GFP intensity at Day 1 and Day 12 of adulthood. Each row of the heatmap represents an individual animal. Each column represents a specific intestinal cell. The identity of each intestinal cell and specific expression level for that cell are listed in S3 Table. The bar graph shows quantitation of average GFP intensity in arbitrary units over all intestinal cells at each age. Error bars indicate SEM. ELT-2:GFP levels decline from Day 1 to Day 12 (p < .05, Student’s t-test). (D) N2 adults fed *elt-2* RNAi starting at Day 1 of adulthood have a lifespan reduced by 50% relative to vector-fed control animals. This experiment was performed twice; one assay is shown. Both assays reached statistical significance as determined by a Log Rank test. (E) *elt-2* overexpression extends lifespan by ~20% relative to transgenic *unc-119(+)* controls. This experiment was performed five times across two lines; one assay is shown. Four of five assays reached statistical significance as determined by a Log Rank test. Data for other assays are in S4 Table.

One explanation for the overlap between ELT-2 GATA targets and age-regulated genes is that either the amount or activity of ELT-2 GATA changes with age, leading to changes in expression of its downstream genes. To test this possibility, we quantitated *elt-2* RNA and ELT-2 protein levels in young and old animals. We used qRT-PCR to quantitate *elt-2* mRNA levels in wild-type animals during aging. We first used qRT-PCR to compare *elt-2* RNA levels in young (L4 larval stage) versus old (day 13 of adulthood) wild-type hermaphrodites. We used six reference genes (*let-70*, *tbb-2*, *htz-1*, *pmp-3*, Y45F10D.4, *cdc-42*) as controls, and observed, on average, a two-fold reduction in *elt-2* expression in day 13 adults (S1 Fig). We then repeated the qRT-PCR experiment to measure *elt-2* expression at L4, day 3, day 6, day 9, and day 12 of adulthood using only *tbb-2* as a control, and observed a two-fold reduction in *elt-2* levels by day 3 of adulthood (Fig 2A).

To quantitate ELT-2 protein levels during aging, we used a worm strain carrying an ELT-2:GFP translational reporter to measure expression during aging by fluorescent microscopy. We used the modENCODE strain that carries integrated copies of the full-length genomic *elt-2* gene with GFP inserted in the 3’ end of the coding region. We observed a 50% decline in GFP fluorescence between Days 1 and 11 of adulthood (Fig 2B). To control for the possibility that expression differences between young and old animals were due to silencing of the *elt-2*:*gfp* transgene in old age, we repeated this experiment using a strain that was homozygous for *rde-1(ne300)*. *rde-1* encodes an Argonaute protein that is required for RNAi interference and the silencing of transgenes [20]. In the *rde-1(ne300)* mutant background, we observed a similar decline in ELT-2:GFP fluorescence during aging, indicating that *elt-2*:*gfp* decreases expression due to aging rather than transgene silencing (S2 Fig).

We also measured changes in ELT-2:GFP expression at the level of single cells using an automated *C*. *elegans* lineage analyzer [21]. For the cell lineage experiments, we first obtained high-resolution three-dimension confocal data stacks of individual worms, aged 1 and 12 days. For each image stack, the cell lineage analyzer first computationally straightened the worm and then identified each intestinal nucleus based on the invariant cell lineage. GFP fluorescence was measured from the entire volume of each nucleus and expression results are presented as a heat map where the color intensity corresponds to expression level in a single nucleus. We used the automated cell lineage analyzer to quantitate ELT-2:GFP levels in the 34 individual intestinal cells in Day 1 and Day 12 adult animals. While just 9 of the intestinal cells showed a statistically significant difference in ELT-2:GFP between Day 1 and Day 12, 26 out of 34 intestinal cell nuclei had less GFP fluorescence in old age (S3 Table). We combined the fluorescence measurements for all of the intestinal cells at each time point and found that ELT-2 GFP decreases by approximately 50% from Day 1 to Day 12 (p < 10^−9^)(Fig 2C). In summary, our results indicate that both *elt*-2 RNA and protein levels decrease during aging.

Declining levels of *elt-2* activity in old age might be detrimental and limit lifespan, might have no effect on worm physiology, or might be beneficial and help promote lifespan. In order to distinguish between these possibilities, we determined the lifespan of animals fed *elt-2* RNAi or overexpressing *elt-2*. The lifespan phenotypes we find from both the *elt-2* RNAi and *elt-2* overexpression strains are consistent with previous reports [16,18]. We reduced *elt-2* activity using RNAi beginning at Day 1 of adulthood and measured lifespan in two independent experiments. To confirm knockdown of *elt*-2, we performed qRT-PCR on worms that had been fed RNAi against *elt*-2, and observed >90% reduction in *elt-2* RNA levels (S3A Fig). We observed a 25% reduction in the median lifespan and a 50% reduction in the maximum lifespan relative to animals fed a control vector (log-rank p< 10^−5^ in each experiment)(Fig 2D). We also measured the lifespan of animals overexpressing *elt-2* relative to transgenic controls. We generated two independent lines containing multiple copies of *elt-2*:*gfp* in an extrachromosomal array (SD1963 and SD1964, S10 Table). We performed RNA-seq on SD1963 and found the abundance of *elt*-2 mRNA, was about 4-fold higher at both a young and an old timepoint in the overexpressor strain relative to a control strain containing the co-transformation marker *unc-119(+)* alone. We assayed the lifespan of these *elt*-2 overexpressor worms and found that median lifespan was extended 15–25% (p-value = 0.003)(Fig 2E, S4 Table). A previous study observed an increase in the median and maximum lifespan of an *elt-2* overexpressor strain [18]. These results indicate that decreasing levels of ELT-2 expression in old age limit normal lifespan, and that increasing ELT-2 expression extends longevity.

### Genes regulated by ELT-2 GATA are induced during intestinal development and decline during aging

We wanted to investigate the functional implications of reduced expression of *elt-2* in old age. Previous work has described several biological functions for ELT-2 GATA. During embryogenesis, *elt-2* regulates differentiation of the intestine by activating the intestinal gene expression program [14]. During adulthood, *elt-2* activity is required for maximal survival in response to pathogen infection, or for maximal longevity in a *daf-2* mutant background [16,18,22]. One possibility is that, in the adult, ELT-2 has a distinct role in innate immunity or *daf-2*-mediated stress protection, or it could suggest that intestinal functions are indirectly required for immunity and stress protection.

A key issue is whether the decline in ELT-2 expression in old age limits lifespan only due to a loss of innate immunity and stress protection or whether old age also involves a loss of general intestinal functions. This issue is important because there is ample precedent that loss of innate immunity and/or stress protection are involved in aging; for example, *pha-4* and *skn-1* are two transcription factor genes that have roles in stress protection and can extend lifespan [19,23]. By contrast, the idea that loss of general intestinal functions are involved in aging is more novel.

In order to study the downstream functions of ELT-2, we performed a genome-wide analysis of the transcriptional regulatory activity of ELT-2 during the L1 stage to define its functions in establishing and maintaining intestinal functions. We then performed an analogous genome-wide analysis of ELT-2 transcriptional activity in the L4 stage to define its functions in a mature intestine, such as innate immunity and stress protection. By comparing the transcriptional activities of ELT-2 in the L1 and L4 stages, we could determine whether ELT-2 GATA has similar or distinct gene regulatory functions in early development and adulthood. We could then ask how these transcriptional activities change in old age.

To identify genes regulated by *elt-2*, we first created an expression signature of genes that respond to *elt-2* regulation at two timepoints: L1 larval stage and L4 larval stage. A transcriptional profile of *elt-2* mutants animals at the L1 stage had been done previously [24]. We extended and confirmed the previous *elt-2* expression signature in the L1 stage by using RNA-seq to identify genes that require wild-type levels of *elt-2* for proper expression. Specifically, we cultured three biological replicates of L1 stage progeny from worms grown on either *elt-2* RNAi bacteria or empty vector control bacteria, and sequenced 3’ end-enriched RNA-seq libraries. We confirmed knockdown of *elt-2* RNA by using qRT-PCR and by observing that the L1 larvae arrested development (S3B–S3D Fig). We used a rank product method with a false discovery rate of 10%, and identified 162 genes that are differentially-expressed in *elt-2* RNAi worms compared to wild-type at the L1 stage (S5 Table). The *elt-2*-regulated gene list consists of 153 genes with lower expression and 9 genes with higher expression in *elt-2* RNAi worms (S5 Table). These *elt-2* regulated genes are strongly enriched for genes that are directly bound by ELT-2, based on our ChIP-seq data (105/162 genes, p < 10^−60^). We compared our results of expression changes in *elt-2* mutants with the previous results. Of the 162 *elt-2* regulated genes, 101 of these showed condordant two-fold changes expression in the previous study (S4 Fig)[24].

**Fig 3:**
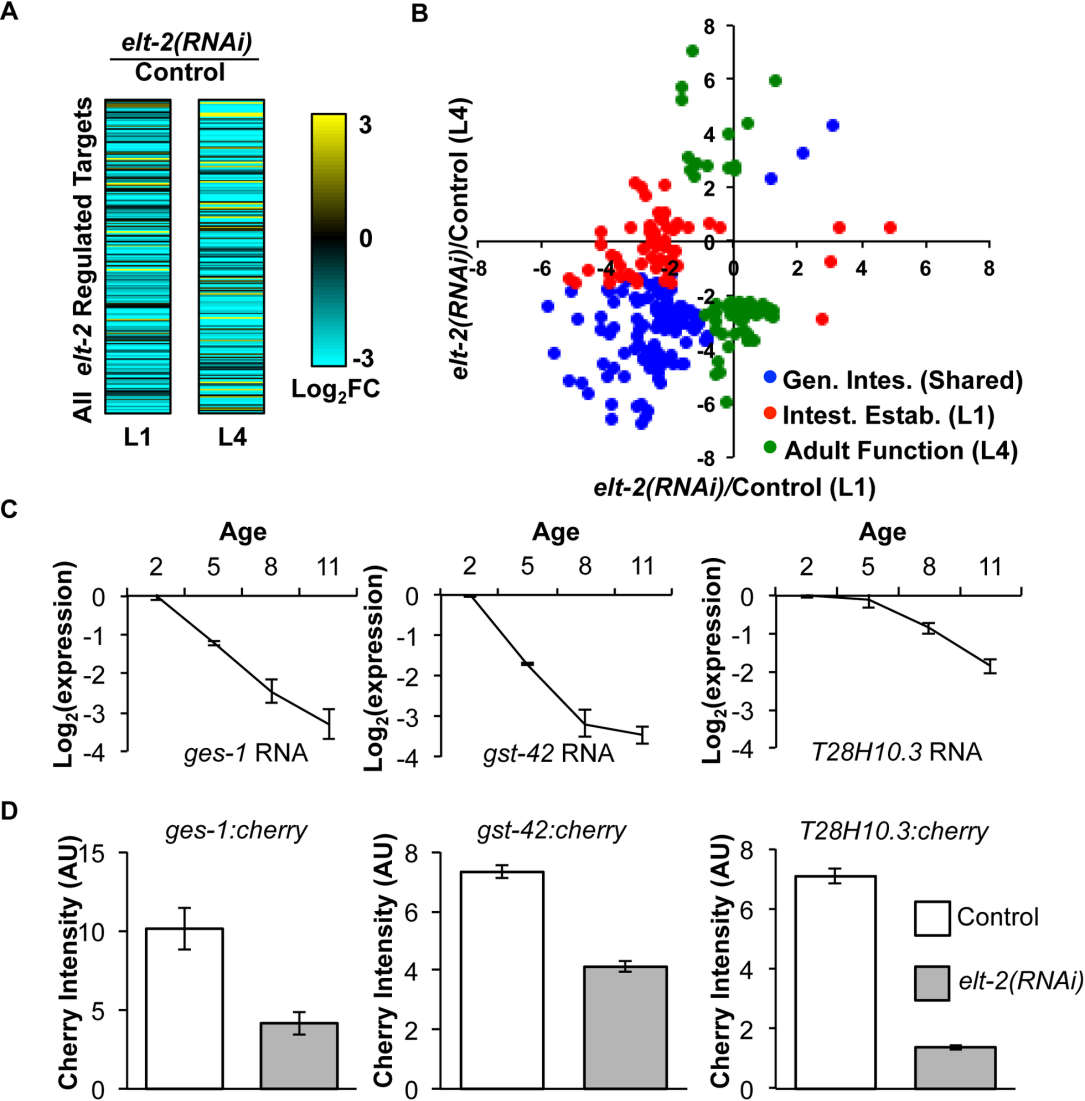
Gene regulation by ELT-2 during development and adulthood. (A) The two heatmaps show log_2_ expression changes between *elt-2(RNAi)* and control animals for the union of the L1 and L4 targets. The left heatmap shows expression changes in the L1 larval stage; the right heatmap shows the expression changes in the L4 larval stage. (B) Scatterplot comparing regulation by ELT-2 of 151 genes in the L1 and L4 stages. X-axis shows log_2_ *elt-2(RNAi)*/control expression ratio at the L1 stage. Y-axis shows log_2_ *elt-2(RNAi)*/control expression ratio at the L4 stage. Red dots indicate genes regulated by ELT-2 GATA only in the L1 stage (intestinal establishment), green dots indicate genes regulated only in the L4 stage (adult function), and blue dots indicate genes regulated in both stages (general intestinal function). (C) Microarray expression data from Budovskaya *et al*. 2008 shows *ges-1*, *gst-42*, and *T28H10*.3 decrease expression during aging (ANOVA, p < 10^−4^). X-axis denotes Days of adulthood. Y-axis denotes log_2_ expression levels normalized to Day 2 of adulthood. Error bars indicate SEM. (D) *elt-2* regulates *ges-1*, *gst-42*, and *T28H10*.3 in adulthood. Animals carrying transcriptional reporters for each of the genes were fed either RNAi against *elt-2* or a vector control on Day 1 of adulthood. Bar graphs show fluorescence intensity measured on Day 3 of adulthood from 40–60 animals per condition. In *elt-2(RNAi)* animals, *ges-1* shows residual expression, possibly due to regulation by *elt-7* (which encodes another GATA factor known to regulate *ges-1* in the intestine) [25]. Error bars indicate SEM.

We identified *elt-2* targets during the L4 larval stage in a similar manner. Synchronized L1 animals were fed either *elt-2* RNAi or vector control, and RNA was isolated at the L4 larval stage. We identified 292 *elt-2*-regulated genes during the L4 stage. We compared the two sets of *elt-2*-regulated genes to each other to evaluate whether gene regulation by ELT-2 during the L1 stage is similar to its regulation in the L4 stage. Fig 3A and Fig 3B display the expression changes of the *elt-2* regulated genes caused by *elt-2(RNAi)* in each stage. In Fig 3B, we used a statistical approach to assign each gene according to whether it was regulated by ELT-2 GATA only in the L1 stage (red dots, referred to as intestinal establishment genes), only in the L4 stage (green dots, referred to as adult function genes) or in both stages (blue dots, referred to as general intestinal function genes)(see Methods and S6 Table for more detail). Out of a total of 223 genes, 103 genes are regulated by *elt-2* at both the L1 and L4 larval stages (general intestinal function class, 46%), 61 genes are regulated only at the L1 stage (intestinal establishment, 27%) and 59 genes are regulated only at the L4 stage (adult intestinal function/innate immunity/stress response, 26%).

Together, the general intestinal function and intestinal establishment categories define a set of *elt-2* regulated genes that are activated by *elt-2* in the intestine starting in early development. There is also a class of genes that are regulated by *elt-2* just in the L4 stage that may have adult-specific functions, such as innate immunity and stress protection. By characterizing which class(es) of *elt-2* genes change expression during normal aging, we will better understand which function(s) of ELT-2 are compromised in old animals.

In addition to a genome-wide analysis, we used mCherry reporters to examine expression of three ChIP-seq targets of ELT-2 GATA from the L1 and L4 larval stages. We selected three genes (*ges-1*, *gst-42*, and *T28H10*.3) that are directly bound by ELT-2 at the L1 stage and are known to be regulated through GATA sites during embryonic and the L1 stage during development (S1 Table)[14,26,27]. By analyzing expression data from DNA microarray experiments from Budovskaya *et al*. 2008, we found that each of the three genes also decrease expression during aging (Fig 3C)[5]. Next, we examined expression of mCherry transcriptional reporters for these genes following *elt-2* RNAi treatment of Day 1 adults. All three transcriptional reporters showed decreased levels of expression in *elt-2(RNAi)* worms compared to controls, in agreement with the DNA microarray results (p < .05, Student’s t-test). These results show that ELT-2 regulates these three genes during both adulthood and development (Fig 3D). These results with reporter genes combined with the genome-wide expression results indicate that a significant portion of ELT-2 regulation is shared between early development and adulthood.

### Reduction of the ELT-2 developmental program during aging

We wanted to understand the impact of the age-related decline in ELT-2 expression. One possibility is that the decline in ELT-2 expression in old age only impacts the expression of the L4-specific genes (adult function), which might suggest that the role of *elt-2* in old age is limited to stress protection and innate immunity. Another possibility is that the age-related decline in ELT-2 expression affects the general intestinal genes, which would suggest that the loss of *elt-2* expression in old age also leads to defects in general intestinal and intestinal establishment functions. To test these possibilities, we profiled gene expression following: 1) *elt-2(RNAi)*, 2) during development (using modENCODE RNA-seq data from seven timepoints during development) and 3) during aging (using data from a DNA microarray timecourse)[5,28].

**Fig 4:**
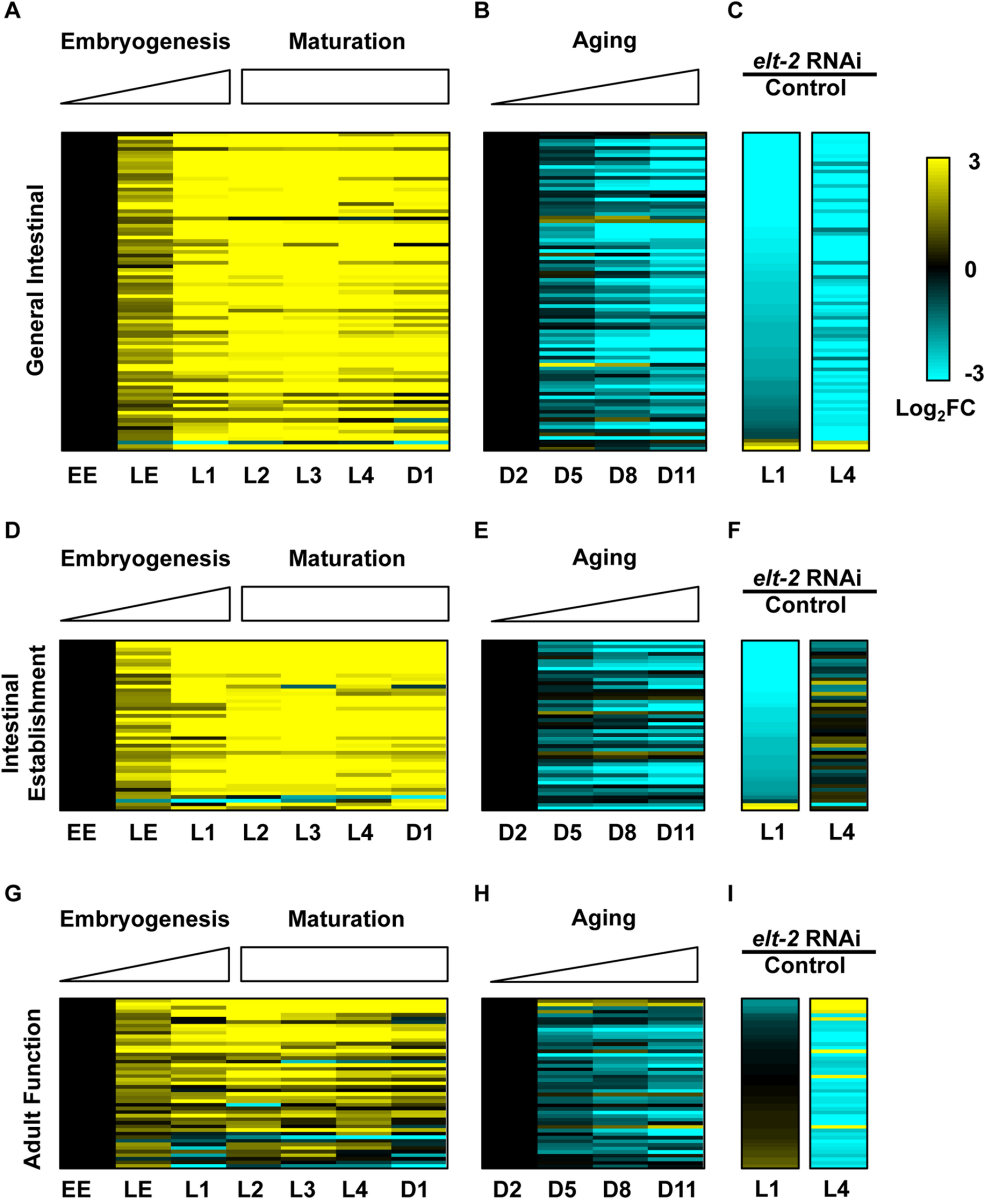
Comparison of expression of *elt-2*-regulated genes during development and aging. For each class of *elt-2*-regulated genes(general intestinal function, intestinal establishment and adult function), the heatmaps show how the genes are expressed during development and aging. Only genes profiled in all three experiments are shown. (A-C) General intestinal genes. (A) Heatmap shows expression changes of the general intestinal genes during development using RNA-seq data from Gerstein *et al*., 2014. Values are Log_2_ expression fold changes during normal development relative to early embryos. Timepoints are early embryo (EE), late embryo (LE), L1, L2, L3, L4, and Day 1 of adulthood. (B) Heatmap showing expression of the general intestinal genes of ELT-2 during aging, using DNA microarray data from Budovskaya *et al*. 2008. Values are Log_2_ expression fold changes during aging relative to Day 2. Timepoints are Days 2, 5, 8, and 11 of adulthood. (C) Heatmaps showing expression of the general intestinal genes following *elt-2* RNAi in the L1 and L4 larval stages. Values are log_2_ fold change in *elt-2* RNAi compared to control RNAi. (D-F) Intestinal establishment genes. Similar to (A-C). (G-I) Adult function genes. Similar to (A-C).

Fig 4 shows the results for the set of ELT-2 general intestinal function genes. As expected, this set shows a near-uniform pattern of activation during development; 93% of these genes increase expression between early embryos and Day 1 Adult worms (Fig 4A).

During aging, 93% of the general intestine genes shared between the L1 and L4 stages decrease expression; the average general intestinal function gene decreases expression by over 5-fold between Day 2 and Day 11 of adulthood (Fig 4B). We accounted for the possibility that these results might be caused by changes in the mass of the intestine relative to the mass of the worm; specifically, if the relative size of the intestine either increased during development or decreased with age, then the results for this gene set may be a property for any intestinal-expressed gene. To exclude this possibility, we used a list of intestinal-expressed genes from Pauli *et al*. 2006 and found that they showed smaller changes in expression during development and aging compared to the general intestinal genes (S5A and S5B Fig, K-S test p-value<10^−16^ in each case) [26].

Next, we examined the age-related changes in expression for the adult function genes and the intestinal establishment genes and observed that expression of both sets of genes decline during normal aging (Fig 4D–4I). While all three sets of ELT-2 genes decline during aging, the expression changes of the adult function genes are less extreme than those of the general intestinal function genes (K-S test p-value = 3.7x10^-3^). In summary, we used the transcriptional output of ELT-2 to define three sets of genes representing establishment of the intestine, general intestinal functions and adult function, and found that all three sets decline during aging.

The changes in expression of the ELT-2 regulated genes in old age resemble their changes following *elt-2(RNAi)*(Fig 4B and 4C). Specifically, 81/88 of general intestinal genes show concordant expression changes following *elt-2(RNAi)* and during aging (92%; p = 2.05x10^-17^). This observation indicates that *elt-2(RNAi)* recapitulates part of the normal aging signature by causing gene expression changes in young animals that resemble changes in old animals. Further, this observation indicates that part of the normal aging process involves loss of ELT-2 GATA transcriptional activity.

### Attenuated transcriptional changes caused by overexpression of ELT-2

**Fig 5:**
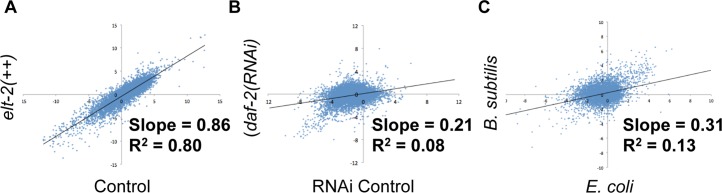
*elt-2* overexpression attenuates gene expression changes during aging. (A) Scatterplot depicts Log_2_ fold change during aging in *elt-2* overexpressing animals and in control animals. Each point represents a single gene, whose X-coordinate is given by its fold change in control animals between L4 and Day 13 of adulthood, and whose Y-coordinate is given by its fold change in the *elt-2* overexpresssing strain between L4 and Day 13 of adulthood. Data are from one of five replicates. Shown are genes with a read depth of at least 10 in either control or *elt-2* overexpressing animals. A best-fit line was generated by linear regression. (B) Scatterplot depicts Log_2_ fold changes during aging (Day 8 of adulthood versus Day 1 of adulthood) in control animals and in *daf-2* RNAi animals. Data are from Murphy *et al*. 2003. (C) Scatterplot depicts Log_2_ fold changes during aging (Day 13 versus Day 4) in animals fed *B*. *subtilis* and in control animals fed standard *E*. *coli*. Shown are the slope and square of the correlation coefficient (R^2^) of the linear regression. Results from all five replicates are shown in S4 Table.

To evaluate the effects of *elt-2* overexpression, we compared the expression changes during aging in control animals to expression changes during aging in an *elt-2* overexpressing strain. If *elt-2* overexpression slows expression changes during aging, we should observe a reduced magnitude of expression changes in the *elt-2* overexpression strain relative to the control strain. In our RNA-seq experiments, we obtained adequate read depth to calculate expression for about 7000 genes. We compared their changes in expression during aging in the control and *elt-2* overexpressing strains. We performed a linear regression analysis of the expression changes and determined the slope of the resulting best-fit line, which provides a comparison of the age-regulated changes across the entire transcriptome for the two strains. We performed this analysis individually for each of the five biological replicates, and found that the *elt-2* overexpressing line had smaller age-related expression changes than the control strain in all five cases (p < 10^−8^ for each regression model)(S7 Table). When averaged together, the magnitude of age-related expression changes from the five replicates was lower in the *elt-2* overexpressing line than in the control strain (average = 0.87, p = .04, 95% confidence interval 0.79–0.96) (Fig 5A, S7 Table). This indicates that overexpression of *elt-2* reduces the magnitude of the expression changes that normally occur over time, suggesting that *elt-2* overexpression causes transcriptional changes that occur during normal aging to proceed more slowly.

To understand the significance of this result, we performed the same linear regression analysis on transcriptome data from young and old worms from two other conditions that extend lifespan: *daf-2* RNAi and *B*. *subtilis* diet (Fig 5B and 5C)[29,30]. For *daf-2* we used data from a gene expression timecourse on both *daf-2* RNAi and vector control [31]. For *B*. *subtilis*, we used data from young and old animals fed *B*. *subtilis*, compared to worms fed standard OP50 *E*. *coli*. Unlike *elt-2* overexpression (average R^2^ = 0.74), we found that age-related transcriptional changes for each of these conditions showed little similarity (*daf-2* R^2^ = 0.08, and *B*. *subtilis* R^2^ = 0.13) to those observed during normal aging, suggesting that the *daf-2* mutation and *B*. *subtilis* feeding extend life by inducing different gene expression pathways rather than slowing down the expression changes that normally occur during aging.

Next, we examined whether there was coordination between different tissues to slow gene expression changes in the *elt-2* overexpressing strain. One possibility is that the effects of ELT-2 overexpression are limited to the intestine, which is the only tissue where it is expressed. Another possibility is that ELT-2 overexpression slows gene expression changes in the intestine, which in turn affects the rate of expression changes in other tissues as well. In order to distinguish between these possibilities, we analyzed age-related expression changes for genes specific to five tissues (S8 Table)[26,32–34]. As above, we generated scatterplots for tissue-specific genes showing the magnitude of their age-related changes in expression in the *elt-2* overexpressing compared to the control strain. As might be expected, the average slope of the regression line for the intestine-specific genes was 0.85, indicating an attenuation of age-related changes in this tissue (p = 0.02, S7 Table). For genes expressed specifically in the muscle, hypodermis, and neurons, the slopes of their best-fit linear regression were also less than one, in all five replicates (S7 Table). Age-related changes in the germline were not significantly affected by *elt-2* overexpression. These results suggest that overexpression of *elt-2* in the intestine influences gene expression in the muscle, hypodermis and neuronal cells as well.

### Regulation of *elt-2* GATA expression

It could be that other transcription factors that bind age-regulated genes from our initial screen also change activity or expression over time. If these factors also regulate *elt-2*, their changing activities could account for the observed decrease in ELT-2 levels in old worms. In order to determine if these other factors regulate *elt-2*, we fed RNAi against six of the other hits from our screen to animals on Day 1 and examined expression of ELT-2:GFP on Day 2 of adulthood and expression of *elt-2* mRNA on Days 2 and 4 of adulthood. We performed fluorescent imaging and compared ELT-2:GFP intensity in these animals to animals fed an RNAi control. *elt-2* mRNA levels were not affected by RNAi against any of the transcription factor genes, and only RNAi against *unc-62* resulted in a change in ELT-2:GFP intensity. These results suggest that ELT-2 expression may be regulated by UNC-62 Hox, but not by the other five transcription factors (S6 Fig).

*unc-62* encodes a Hox cofactor that is differentially spliced such that the *unc-62A* isoform appears exclusively in the intestine [9]. We found that UNC-62 binds to the upstream region of *elt-2* by analyzing ChIP-seq data from the modENCODE consortium (S7 Fig). Furthermore, *elt-2* expression increases in *unc-62(RNAi)* worms, indicating that UNC-62A normally functions to repress *elt-2* expression in the intestine (Table 1). These results show that UNC-62A directly regulates *elt-2*. However, as worms age, expression of UNC-62A in the intestine decreases, which should cause *elt-2* expression to increase with age, opposite to what is observed [9]. Additionally, expression of ELT-2:GFP still decreases during aging when worms are fed RNAi targeting *unc-62* (S8 Fig). Hence, factors other than UNC-62A are likely to be responsible for the decrease in *elt-2* expression in old age.

**Table 1:** Lifespan mutants have increased levels of *elt-2* expression.

Genotype^a^	Control	Age^b^(Temp)	Assay	*elt-2* FC^c^	p-val
*unc-62* RNAi^a^	Vector	Day 4 (20°C)	GFP	**3.9**	1.4E-17
*AmpK* OE	*unc(+)*	Day 13 (20°C)	qRT-PCR	4	5.8E-02
*hsf-1* OE	*unc(+)*	Day 13 (20°C)	qRT-PCR	**3.2**	2.0E-04
*lmp-2* OE	*unc(+)*	Day 13 (20°C)	qRT-PCR	**2.6**	6.8E-03
*D*. *rerio sod-1* OE	*unc(+)*	Day 13 (20°C)	qRT-PCR	**2.7**	5.0E-02
*clk-1*	N2	Day 10 (25°C)	qRT-PCR	**1.7**	5.0E-03
*daf-2*	N2	Day 10 (25°C)	qRT-PCR	**1.9**	7.0E-03
*glp-1*	*glp-4*	Day 10 (25°C)	GFP	**5.6**	9.6E-08
**Dietary Restriction**					
*eat-2*	N2	Day 10 (25°C)	qRT-PCR	**0.77**	5.0E-02
N2 (sDR)	N2 (AL)	Day 10 (25°C)	qRT-PCR	0.88	5.9E-01

^a^Descriptions of mutations and references for extended lifespans can be found in S9 Table.

^b^Days of adulthood.

^c^*elt-2* Fold Change. Ratio of *elt-2* levels in long-lived condition to control.

We also investigated regulation of *elt*-2 expression during aging by ELT-7, which is another intestinal GATA transcription factor. During embryogenesis, ELT-2 and ELT-7 are known to perform similar function in regulating intestinal gene expression, and ELT-7 is known to promote expression of *elt-2* [25]. To test whether ELT-7 GATA regulates *elt-2* expression in adult animals, we performed RNAi against *elt-7* in Day 1 Adult animals followed by qRT-PCR of *elt-2*. As a control, we found that *elt-7(RNAi)* was effective in reducing the level of *elt-7* mRNA (S9 Fig). However, *elt-7(RNAi)* did not alter the abundance of *elt-2* mRNA in adult animals (S9 Fig). We also compared the lifespan of *elt-7(RNAi)* animals to control animals, and found no difference in lifespan (S9 Fig). These results indicate that *elt-7* is unlikely to be responsible for the declining expression of *elt-2*, and that *elt-7* is dispensable for normal lifespan.

### Increased expression of *elt-2* is a common feature of long-lived mutants

The results presented above show that low levels of *elt-2* in normal old age is harmful and limits lifespan. Hence, in order for a worm to show extended longevity, it may either increase the expression of *elt-2* or alter the worm’s physiology to protect it from the harmful effect of low *elt-2* levels. We selected eight mutant strains that achieve longevity through different cellular mechanisms and compared levels of *elt-2* expression in old animals in the long-lived mutant and controls (S9 Table). For six longevity strains (*daf-2*, *clk-1*. AMP Kinase, *hsf-1*, *lmp-2*, and *Danio rerio sod-1*), we compared *elt-2* mRNA levels by qRT-PCR in mutant animals to control animals of the same chronological age. For *glp-1(e2141)* and *glp-4(bn2)* mutants, we measured levels of ELT-2:GFP protein expression.

We observed significantly increased levels of *elt-2* expression during adulthood in seven of the eight strains (*daf-2*, *clk-1*, *glp-1*, *hsf-1* OE, *lmp-2* OE, *sod-1* OE and *unc-62A* RNAi)(Table 1). For the eighth strain (expressing an activated form of AMP kinase), there was a four-fold increase in *elt-2* expression that was borderline significant (p = .057). These eight mutants affect diverse pathways and extend lifespan by different mechanisms, implying that most lifespan mutants result in increased expression of *elt-2*.

We also examined late-life *elt-2* expression levels in dietary restriction conditions (Table 1). Dietary restriction extends worm lifespan but also directly affects the size and physiology of the intestine. We used two dietary restriction interventions known to extend lifespan: 1) *eat-2(ad1116)* mutants have low rates of pumping causing them to eat less, and 2) the solid dietary restriction method (sDR), which involves feeding worms diluted levels of *E*. *coli*. We assayed *elt-2* levels by qRT-PCR in old worms (Day 13 of Adulthood) under dietary restriction and *ad libidum* feeding conditions. We detected 23% less *elt-2* mRNA in *eat-2* animals compared to wild-type controls (p = .05), but no significant difference between sDR and control animals. Under dietary restriction conditions, intestinal function is reduced from lack of feeding and may also lead to lower expression of *elt*-*2*. Dietary restriction may activate an alternate pathway that allows animals to achieve extended life in spite of low *elt-2* levels. In summary, lifespan extension for nearly all of the long-lived mutants examined involves increased levels of *elt-2* expression in old age, and lifespan extension via dietary restriction involves inducing pathways that act independently of normal intestinal function.

## Discussion

### The role of ELT-2 in gene expression changes during aging and in limiting lifespan

**Fig 6:**
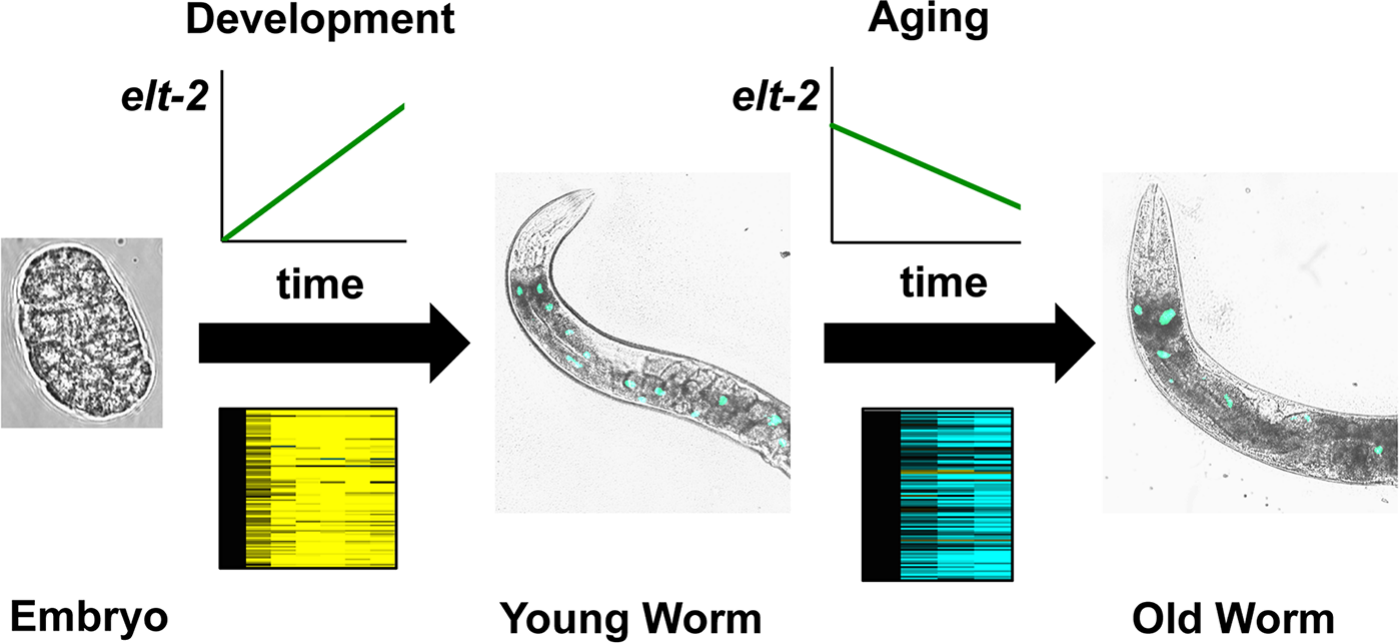
Model for transcriptional control of aging. Following an unbiased screen of transcription factors profiled by the modENCODE project, we identified the ELT-2 GATA transcription factor as a major regulator of RNA changes during aging. *elt-2* is activated during embryonic development, and activates a gene expression program to properly differentiate the intestine. Early in adulthood, *elt-2* expression begins to decline, initiating a network of transcriptional changes that limit lifespan. Loss of activity of *elt-2* is part of the mechanism that limits normal lifespan.

Transcriptional profiling is a powerful way to study aging because it characterizes molecular changes during aging in a quantitative and unbiased way. We utilized the modENCODE ChIP-seq database to perform an unbiased screen for candidate regulators of the aging transcriptome. Using this approach, we found that the factor with the highest overlap was the well-studied developmental transcription factor *elt-2*, implicating it as a transcriptional regulator of genes that change during aging. In this work, we found that declining levels of ELT-2 GATA in the intestine lead to reduced expression of intestinal genes during normal aging and a limited lifespan (Fig 6). ELT-2 is known to activate a large set of genes in the intestine that perform general functions in the gut. We defined the genes regulated by ELT-2 during gut development and found that they show a general decline in old age. This result indicates that part of the aging process involves a general decline in intestinal functions in *C*. *elegans*.

Overexpression of ELT-2 extends lifespan, and lessens the magnitude of changes to the normal aging transcriptome as the worm transitions from young to old. For the ELT-2 overexpressing strain, worms with an old chronological age have transcriptional profiles that would be expected of worms of a younger chronological age, implying that ELT-2 overexpression slows down the rate of aging itself. By contrast, animals fed *daf-2* RNAi or *B*. *subtilis* have an increased lifespan but dramatically altered gene expression profiles during aging. Changes in the transcriptome during normal aging bear little resemblance to the transcriptional changes in either of these conditions that extend lifespan.

*elt-2* is expressed exclusively in the intestine, yet the effects of *elt-2* overexpression occur in other tissues, as well. This is evidence of coordination of aging between tissues, such that slowing aging in a single tissue (i.e. the intestine) can propagate the effect to other parts of the organism, either through signaling or through a different mechanism, such as better nutrition to the rest of the organism.

### Upstream regulators of ELT-2 expression during aging

The upstream factors responsible for the decline in ELT-2 GATA expression during aging are unknown. During embryonic development, expression of *elt-2* is directly activated by several transcription factors including END-1, END-3, and ELT-7 [14,25,35,36]. END-1 and END-3 directly activate *elt-2* expression [35,36]. However, *end-1* and *end-3* are expressed only for a short window of time during embryogenesis, and not at all during adulthood [35,37,38]. ELT-7 does not maintain expression levels of *elt-2* in adult animals (S9 Fig). Hence, none of these transcription factors appear to regulate *elt-2* expression in adult animals. Once its expression is activated late in embryogenesis, ELT-2 maintains its own expression in an auto-activation loop through direct binding to its own promoter [14,39]. One possibility is that gradual loss of auto-activation during aging could explain the loss of *elt-2* expression in old age.

Another possibility is that changes in other pathways that affect lifespan are responsible for the loss of *elt-2* expression during normal aging. We found that many long-lived strains display increased *elt-2* levels in old age. One or more of these pathways could reduce the expression of *elt-2* over time if the activities of these pathways were to change with age. For *unc-62*, expression of the *unc-62*A isoform in the intestine declines with age rather than increases as would be expected if *unc-62* were solely responsible for age-regulation of *elt-2* [9]. For the other pathways, it is currently unclear how their activities change in old age. The eight long-lived strains examined in this work represent a small subset of the total number of genes known to affect lifespan in *C*. *elegans*. Many other lifespan mutants may also have increased *elt-2* expression, and would then be candidates for upstream regulators responsible for low expression of *elt-2* in old age.

A third possibility is that the decline in *elt-2* expression in old age might be a manifestation of damage accumulation. It is possible that the ELT-2 GATA protein may be sensitive to molecular damage, and that molecular damage of the ELT-2 protein could lead to reduced expression of the *elt-2* gene by loss of auto-activation. However, ELT-2 expression begins to decline as early as Day 3 of adulthood, which is when there are few signs of overt damage or aging. Furthermore, not all transcription factors have reduced activity in old age, and thus it is not clear why oxidative damage would affect ELT-2 activity specifically and not other transcription factors.

The low levels of *elt-2* expression in old age limit lifespan. A variety of mutants and RNAi treatments with extended lifespan show increased levels of *elt-2* expression in old age. Furthermore, loss of *elt-2* activity shortens the lifespan of three long-lived mutants that have been tested: *daf-2* (insulin signaling), *eat-2* (caloric restriction), and *isp-1; ctb-1* (mitochondrial dysfunction)[5,15]. Together, these results indicate that, for a wide variety of mutants and treatments, part of the strategy to increase lifespan involves increasing *elt-2* expression in old age. The intestinal transcriptional network includes genes necessary for digestion, pathogen resistance, and epithelial cell polarity. Since the functions of these intestinal genes are essential for survival, global loss of their activities during the aging process limits lifespan.

### Reduced activity of the intestinal gene expression program limits lifespan

We found that the intestinal gene expression program controlled by the ELT-2 GATA transcription factor is lost during the aging process. This observation is surprising as aging is often thought to involve accumulation of damage rather than global changes in a developmental pathway. Understanding the upstream causes for changes in the ELT-2 transcriptional program might reshape how we think about the aging process.

One model for aging is that damage accumulation serves as a molecular timer setting the pace of age-related changes [40]. According to this model, the age-related decrease in ELT-2 activity could be the result of an accumulation of damage of upstream factors needed to maintain ELT-2 expression. This possibility would reshape our thinking about damage accumulation because its effects would be centered on damage to key regulatory molecules, such as ELT-2, rather than distributed across the entire cellular proteome.

Another possibility is that changes in ELT-2 expression could be due to a loss of homeostatic control of expression, a molecular timing mechanism that might act independently of damage accumulation. Many transcription factors regulate the level of ELT-2 expression from embryogenesis to adulthood. For instance, three GATA factors (END-1, END-3, and ELT-7) and autoregulation by ELT-2 control *elt-2* expression during embryogenesis [14,25,35,36,39]. The Hox cofactor UNC-62A sets the level of *elt-2* expression in the young adult. Just as the control of developmental ELT-2 expression is set by these regulatory factors independently of damage, changes to ELT-2 expression during aging could also occur independently of damage.

There has been a considerable effort to understand whether the reproducible changes observed in old animals constitute a program for aging (reviewed in [41]). During development, activation of the ELT-2 gene expression program is under strong evolutionary pressure to establish intestinal fate and function in the growing worm. In contrast to ELT-2’s role during development, changes in ELT-2 expression in old age are not likely to affect evolutionary fitness and are not likely to have evolved by natural selection. This is because the collapse of the ELT-2 transcriptional network that occurs after Day 3 of adulthood is after the time when most nematodes have died in the wild [42]. Hence, the consequent transcriptional changes seen in old age are rarely seen in nature, are probably outside the force of natural selection and are not a program with an adaptive function for aging (reviewed in [41]). During aging, changes in expression of the genes regulated by ELT-2 appear ordered not because they evolved for age-related changes. Rather, they appear ordered because changes in expression of an upstream regulator such as ELT-2 (even if not evolutionarily selected) are passed along to its downstream genes.

An emerging theme in aging research is the recognition that regulatory factors that initially act during development often change abundance during aging. Besides ELT-2 GATA in *C*. *elegans*, other developmental factors that change activity in old age include UNC-62 (Homothorax), the ELT-3, ELT-5, and ELT-6 pathway of GATA transcription factors, and PQM-1 (Zn Finger) [5,9,10]. This type of aging misregulation is also described in mice. The Wnt signaling pathway and p16 (a cell cycle repressor) are developmental regulators that change with age and are partly responsible for age-related degeneration in many organ systems [43–46]. The general principle of aging promoted by drift of developmental pathways in old age is seen in multiple species, although the specific pathways are different in different organisms. These examples illustrate an emerging view of aging that involves alterations of regulatory programs in old age that act previously in young animals to establish developmental pattern and organ function.

## Methods

### ChIP-seq data acquisition and analysis

ChIP seq data were acquired from modENCODE (www.modencode.org). modENCODE produced ChIP seq data by immunoprecipitating GFP from a line expressing ELT-2:GFP that was stably integrated into the genome. The ELT-2 ChIP-seq data set included two biological replicates at the L1 larval stage, using controls and protocols previously described [47]. Significant peaks were called using the PeakSeq algorithm with a threshold of q < 10^−5^ [48]. Only peaks that were identified in both biological replicates were considered for analysis. Peaks were mapped to genes if the position of maximum read density was within the 5 kilobase pairs upstream of the gene’s annotated transcription start site, or contained within the gene body, as previously described [13]. The ELT-2 ChIP-seq dataset was not initially released by modENCODE because it did not meet one of the data quality thresholds–that the top 40% of peaks have a 70% correlation between replicates. The ELT-2 data were validated in this work by showing that intestinally-enriched genes are overrepresented among ELT-2’s ChIP-seq targets, consistent with its known function in intestinal development (S10 Fig).

modENCODE generated ChIP seq data for 58 transcription factors in 99 datasets (some transcription factors were profiled at multiple developmental time points). All 99 ChIP-seq datasets produced by modENCODE were analyzed for binding to the list of age-regulated genes from Budovskaya et al 2008. As in Van Nostrand *et al*., 2013, only low-complexity ChIP-seq peaks (fewer than 8 other factors bound) were considered for the analysis. Significance of overlap was calculated using a Chi-squared test. We accounted for multiple hypothesis testing using a Bonferroni-corrected significance threshold of p = .05/99 = 5x10^-4^.

### Worm culture conditions

Lifespans and aging timecourses were performed on NGM plates containing 30 mM FUDR. Worms were moved to FUDR on Day 1 of adulthood (when eggs were present). All experiments were carried out at 20 °C unless otherwise specified. Lifespans were initiated with at least 100 worms in each group. Timecourse experiments were initiated with at least 30 animals per group. Animals with burst vulvas were censored from the experiment. Data for all lifespan experiments can be found in S3 Table. Lifespan statistics were determined with a Log Rank test. Strains and associated details are listed in S10 Table.

### Strains

The ELT-2:GFP strain OP56 was produced by modENCODE for ChIP-seq analysis. Briefly, the strain was constructed by biolistic integration of the fosmid clone 915685980012804 H01 into the *C*. *elegans* genome [49]. To simplify image analysis, we constructed a strain (SD1949) that is homozygous for the ELT-2:GFP transgene (Is*(elt-2*:*gfp*) and also homozygous for *glo-4(ok623)*, which has less gut autofluorescence.

The *elt*-2:*gfp* strain generated by modENCODE is unsuitable for lifespan analysis because it contains an integrated fosmid (clone 915685980012804 H01) containing 3 other genes in addition to *elt-2* (C33D3.3, C33D3.4, C33D3.5). A lifespan phenotype for this strain could be due to the effect of any one, or several, of the genes in the fosmid. For lifespan analysis, we generated new *elt-2* overexpression lines: SD1964 and SD1965. We used Gateway cloning (Thermo-Fisher Scientific) to generate a plasmid containing 5 kb of DNA directly upstream of *elt-2*, the *elt-2* coding region fused to GFP, and followed by the *unc-54* 3’UTR. This plasmid contains a functional copy of *unc-119* to facilitate the selection of transgenic animals. A previous report showed that the expression of an *elt-2* transgene is increased in an *rde-1* mutant background [50]. We injected the above plasmid into the germline of *unc-119(ed3);rde-1(ne300)* animals. We isolated two lines of stably-transmitting *unc-119(+)* animals which carry multiple copies the *elt-2*:*gfp* plasmid as an extrachromosomal array (SD1964 and SD1965). To create a control strain, we first generated a similar plasmid lacking the *elt-2* promoter, coding sequence, or GFP, but containing the *unc-119(+)* positive selection marker. We injected that plasmid into the germline of *unc-119(ed3);rde-1(ne300)* animals and isolated a line of stably-transmitting *unc-119(+)* animals resulting in the control strain SD1965.

To generate strains expressing ELT-2:GFP that lack a germline, we crossed strain SD1949 to two strains of animals carrying temperature-sensitive mutations: *glp-1(e2141)* and *glp-4(bn2)*. The cross was carried out at 15°C to prevent sterility of the animals. F2 progeny were singled to individual plates. Worms were transferred after the first day of adulthood to new plates to produce 2 plates of F3 progeny from each F2. For each F2, one of the two plates of F3 animals was transferred to 25°C to screen for homozygosity of the germlineless allele. Homozygosity of the *elt-2*:*gfp* transgene was confirmed by microscopy.

The transcriptional reporter strains SD1429 (*gst-42*:*Cherry*), SD1960 (*ges-1*:*Cherry*), and RW10819 (*T28H10*.*3*:*Cherry*) were previously generated. For *gst-42*, *ges-1*, and T28H10.3, the promoter constructs from the *C*. *elegans ‘*Promoterome’ (http://worfdb.dfci.harvard.edu/promoteromedb/) were inserted by Gateway cloning into an expression vector containing the Histone 1 coding region fused to Cherry and *unc-119(+)* to facilitate screening for transgenic animals. These plasmids were integrated into the genome by biolistic bombardment.

Strains SD1821 (*lmp-2* overexpression), SD1822 (*hsf-1* overexpression), SD1823 (*aakg-2* overexpression), and SD1829 (*sod-1* overexpression) were described in [51].

### qRT-PCR

qRT-PCR was used to measure transcript levels. At least 30 worms were used for each of at least three biological replicates. Total RNA was extracted from worms using TRIzol reagent (Life Technologies) and phenol-chloroform extraction. Single-stranded cDNA was prepared using oligo-dT primers. qRT-PCR primers for *elt-2* were designed to span splice junctions to prevent amplification of genomic DNA. Before use in qRT-PCR experiments, each oligonucleotide pair was confirmed to amplify only cDNA, with no detectable amplification of genomic DNA. Three technical replicates were performed for each sample. Similarly designed primers against *let-70*, *tbb-2*, *htz-1*, *pmp-3*, Y45F10D.4 or *cdc-42* were used as references. qRT-PCR was performed using the Applied Biosciences SYBR Green PCR Master Mix and following the protocol described in Zimmerman *et al*., 2014 [52].

We tested our reference primers to measure *elt-2* levels in young (L4) and old (Day 13) worms. At the outset, we did not know of a specific control gene that did not change with age. If expression of the reference gene were to change with age, then calculation of the expression level of *elt-2* would be affected. We initially used six different reference genes as controls (*let-70*, *tbb-2*, *htz-1*, *pmp-3*, Y45F10D.4, *cdc-42*), none of which were previously thought to change expression with age. We found that the change in the abundance of *elt-2* from L4 to Day 13 was similar when each of the six references were used individually, suggesting that none of the reference genes are strongly age-regulated. For subsequent qRT-PCR analyses, we used *tbb-2* as a reference gene.

*elt-2* was not identified as being age-regulated in a previous DNA microarray experiment [5], possibly because the level of *elt-2* RNA is relatively low or because DNA microarray experiments are relatively noisy.

### Fluorescence imaging

Worms were mounted on slides with a pad of 2% agarose. A solution of 100μg/mL levamisole was used to immobilize the worms. A coverslip was placed on the slide, and worms were immediately imaged on a Zeiss Axioplan compound microscope under the 20X objective. Exposure times were selected such that no image reached saturation intensity. For each imaging experiment, images for all timepoints/conditions were acquired on the same day using the same microscope and camera settings so data can be fairly compared. When possible, the entire worm was imaged in a single field, otherwise, it was imaged in two overlapping fields.

ImageJ was used to quantitate integrated fluorescence intensity in the intestinal nuclei. For each worm, two background measurements adjacent to the intestinal nuclei were measured. These background measurements were averaged and subtracted from each integrated intensity measurement. Several strains carried the *glo-4(ok623)* mutation to reduce intestinal autofluorescence. See Strain List for details.

### Cell-lineage analysis

Lineage analysis was performed as previously described on SD1949 [21]. Following computational straightening of image stacks and nuclear segmentation, intestinal nuclei were manually annotated with the VANO program. Background fluorescence was estimated as the average signal of 10 pseudonuclei drawn adjacent to the intestine. The fluorescent signal represents the sum of all GFP signal over every voxel of each nucleus. A previous study characterized the single-cell expression patterns from the automated cell lineage analyzer to the endogenous expression patterns assayed (either by antibody staining against the endogenous transcription factor or in situ hybridization to the endogenous transcription factor mRNA) for 53 low-copy number transgenic lines [21]. In 47/53 cases, there was strong agreement between the expression pattern from the cell lineage analyzer and the endogenous measurement.

### RNA sequencing and analysis

For all transcriptional profiling experiments, at least three biological replicates each of control and experimental were grown. Worms were grown on standard NGM supplemented with 30μM FUDR (young vs. old transcriptome) or 1mM IPTG and 100 μg/mL Ampicillin (RNAi experiments). Culture conditions for each experiment are outlined below. 3’-end enriched RNA-sequencing (3SEQ) libraries were prepared as previously described [9,53]. Briefly, barcoded sequencing adapters were ligated onto cDNA made from poly-A-enriched RNA. Libraries were pooled prior to sequencing.

In all RNA-sequencing experiments, 110–165 million reads were obtained for each pooled sample, corresponding to 30–50 million reads per biological replicate. Significant gene expression changes were identified using the RankProd R Package to implement the analysis, which identifies differentially expressed genes at a 10% FDR cutoff, as in [9]. Because 3SEQ data only sample the 3’end of transcripts, the data are insensitive to gene size. Read counts were normalized for each gene as the fraction of reads obtained for that gene out of all total uniquely-mapped reads. Data have been deposited in GEO. Accession numbers can be found in S11 Table.

#### *elt-2*-regulated gene identification (L1 stage animals)

A synchronized population of approximately 10,000 N2 animals was fed either *elt-2* RNAi or an empty vector control on Day 1 of adulthood. After 24 hours of exposure to RNAi, adult animals were bleached. Embryos were synchronized by hatching overnight in S-Basal. The resulting L1 animals were pelleted and placed into TRIzol. Three biological replicates were collected for each sample. Differentially expressed genes were identified as described above.

#### *elt-2*-regulated gene identification (L4 stage animals)

A synchronized population of approximately 10,000 N2 animals at the L1 stage was placed onto bacteria expressing dsRNA against *elt-2* or an empty vector control. Animals were washed from plates when they reached the L4 stage roughly 28 hours later. The animals were pelleted and placed into TRIzol. Three biological replicates were collected for each sample. Differentially expressed genes were identified as described above.

#### Classification of *elt-2*-regulated genes

General intestinal function genes were identified as follows. For genes that were identified as *elt-2*-regulated at the L1 stage, they must show a concordant and significant expression change following *elt-2(RNAi)* in the L4 stage (p-value < .05). For genes that were identified as *elt-2*-regulated at the L4 stage, they must show a concordant and significant expression change following *elt-2(RNAi)* in the L1 stage (p-value < .05).

#### *elt-2* overexpression transcriptomes

A synchronized population of approximately 10,000 control or *elt-2* overexpression animals was placed onto normal NGM plates spotted with *E*. *coli* strain OP50. Approximately 28 hours later, L4 stage worms were washed from the plates and placed into TRIzol. For aged samples, adult animals were transferred to FUDR-containing plates at Day 1 of adulthood. On Days 2, 3, 4, 5, 7, 9, 11, and 13 of adulthood, worms were washed from plates onto nylon mesh to filter out eggs and potential progeny. On Day 13, dead animals were manually removed from the plates. The remaining animals were washed from the plates, pelleted, and placed into TRIzol. Five biological replicates were collected for each sample. To avoid differential expression of the most highly-expressed genes skewing the relative abundance of other transcripts, the most highly-expressed 3% of genes in each library were excluded from this analysis. The linear regression analysis was performed as follows. First, the log_2_ ratio of Day 13 to L4 larval expression was calculated for each gene, in both the control and *elt-2* overexpressor conditions. A linear regression analysis was performed to calculate a best-fit line to these data. This analysis was performed separately for all 5 sets of replicates, producing 5 separate slopes of the best-fit lines. These five slopes were averaged to produce an estimate of the true value of this parameter.

#### *E*. *coli* vs. *B*. *subtilis*-fed transcriptomes

A synchronized population of approximately 10,000 N2 animals at the L1 stage was cultured on either *E*. *coli* strain OP50 or *B*. *subtilis* strain PY79. Beginning at Day 2 of adulthood, worms were washed onto nylon mesh every other day. Samples were collected for Day 4 and Day 13 of adulthood, and placed into TRIzol. One biological replicate was collected for each sample. Calculation of the aging slope for *B*. *subtilis* and *E*. *coli* fed worms was done as described above.

### Other datasets used in this study

The data from the microarray timecourse that produced the list of age-regulated genes are from Budovskaya *et al*., 2008. The authors provided the data as the log_2_ ratio of cy5-labeled sample to cy3-labeled reference. For our analyses, the expression of each gene across each stage was normalized to its expression at the initial Day 2 timepoint.

Data for *elt-2* null vs. wild-type L1 larvae used Serial Analysis of Gene Expression (SAGE) from McGhee *et al*., 2009. A single biological replicate was collected for each genotype. Results of SAGE were provided as Reads Per Million.

Data for the modENCODE developmental timecourse used RNA-seq from developmentally-staged hermaphrodites and were obtained from Gerstein *et al*., 2014. Three biological replicates were collected for each of seven developmental timepoints. The authors provided the data as RPKM (Reads Per Kilobase of transcript per Million mapped reads). For our analysis of this timecourse, the expression of each gene across each stage was averaged across biological replicates and normalized relative to its expression at the initial Early Embryo (EE) timepoint.

Data for the gene expression profile of *daf-2* RNAi and control animals during aging were obtained from Murphy *et al*., 2003. The linear regression analysis was performed as follows. First, the log_2_ ratio of old Day 8 to young Day 1 expression was calculated for each gene, in both the control and *daf-2* RNAi conditions. A scatter plot was constructed containing the log_2_ ratios for every gene, and a linear regression analysis was performed to calculate a best fit a line to the data.

## Supporting Information

S1 Fig Declining levels of *elt-2* mRNA in old age using six reference genes. We measured levels of *elt-2* mRNA by qRT-PCR in young and old using six different genes as controls. qRT-PCR was performed on mRNA extracted from three biological replicates (with three technical replicates per biological replicate) of young (L4) and old (Day 14) wild-type worms. *elt-2* levels for young and old animals were measured using each reference gene as a control. The young timepoint was normalized to 1. Error bars indicate SEM.
(PDF)

S2 Fig Declining levels of ELT-2:GFP in old age are not due to transgene silencing. Bar graph shows quantitation of average ELT-2:GFP intensity per animal from SD1989. SD1989 contains the ELT-2:GFP fosmid constructed by modENCODE integrated at a low copy number and is homozygous for *rde-1(ne300)*. 10–20 animals were used for each time point. GFP levels were measured by fluorescence microscopy and intensity levels were analyzed by ImageJ as described in Methods. Error bars indicate SEM. ELT-2:GFP levels are lower at Day 7 and Day 12 than at Day 1 (p < .05, Student’s t-test).
(PDF)

S3 Fig Effectiveness of *elt-2* RNAi. (A) Abundance of *elt-2* RNA in adult animals fed dsRNA against *elt-2* or an empty vector control starting at Day 1 of Adulthood, measured by qRT-PCR. *tbb-2* was used as a reference. Error bars indicate SEM from three biological replicates. The abundance of *elt-2* RNA is reduced by *elt-2(RNAi)* (p < .05, Student’s t-test). (B) Abundance of *elt-2* RNA in wild-type controls and L1 larvae that are the progeny of adult wild-type hermaphrodites fed dsRNA against *elt-2* or an empty control vector, measured by qRT-PCR. Error bars indicate SEM from three biological replicates. The abundance of *elt-2* RNA is reduced by *elt-2(RNAi)* (p < .05, Student’s t-test). (C/D) Shown are progeny (3 days after hatching) from adult wild-type hermaphrodites that were fed dsRNA against *elt-2* (C) or against an empty control vector (D). For *elt-2(RNAi)*, approximately 75% of progeny showed larval-arrest.
(PDF)

S4 Fig Similarity of the *elt*-2-regulated genes in two independent studies. The two heatmaps show log_2_ expression changes between *elt-2* loss-of-function and control animals for the *elt-2*-regulated genes at the L1 stage. The left heatmap shows expression changes between *elt-2* RNAi and control-fed animals at the L1 stage, from RNA-seq data produced in this paper (average fold-change in from three biological repeats). The right heatmap shows expression changes between an *elt-2* null mutant and wildtype L1 larvae, from SAGE data from McGhee et al., 2009 (fold-change from a single repeat). Shown is the concordance between the two data sets, defined as genes with expression changes of the same sign in both conditions.
(PDF)

S5 Fig Expression changes of *elt-2*-regulated genes during development and aging. (A) Histogram of gene expression changes during development. Data are presented as the Log_2_ ratio of relative abundance at Day 1 of adulthood compared to early embryos, using RNA-seq data from modENCODE [28]. Three series are plotted: all genes (black), 1618 intestinally-expressed genes from Pauli et al., 2006 (cyan), and 88 *elt-2* General Intestinal genes (red)[26]. The General Intestinal genes tend to be induced during development more than either intestinal-expressed genes or other genes in the genome (Kolmogorov-Smirnov p-value, General Intestinal vs. all genes: 1.7x10^-33^, General Intestinal vs. intestine-expressed genes: 1.8x10^-27^). (B) Histogram of gene expression changes during aging. Data are presented as the Log_2_ ratio of relative abundance at Day 11 of adulthood compared to Day 2 of adulthood, using DNA microarray data from Budovskaya et al., 2008. Three series are plotted: all genes (black), 1618 intestinally-expressed genes (from Pauli et al., 2006), and 75 General Intestinal genes (red) [26]. The levels of the General Intestinal genes tend to decline during aging more than either intestinal-expressed genes or other genes in the genome (Kolmogorov-Smirnov p-value, General Intestinal vs. all genes: 3.2x10^-29^, General Intestinal vs. intestine-expressed genes: 3x10^-17^).
(PDF)

S6 Fig Candidate RNAi screen for regulators of ELT-2:GFP expression. (A) Bar graph shows quantitation of average GFP intensity of SD1949, which carries the modENCODE ELT-2:GFP fosmid. Adult animals were fed dsRNA against the gene sequence corresponding to a given transcription factor at Day 1 of adulthood and GFP expression was measured at Day 2 of adulthood. Values are shown as arbitrary units indicating GFP levels per animal from a population of 10–20 animals. ELT-2:GFP levels are significantly lower following *elt-2(RNAi)* and significantly higher following *unc-62(RNAi)* (* = Bonferroni-corrected p < .05, Student’s t-test). Error bars indicate SEM. (B) Bar graph shows quantitation of average *elt-2* mRNA relative to tubulin. Adult animals were fed dsRNA against the gene sequence corresponding to a given transcription factor at Day 1 of adulthood. Samples of 20–30 worms were collected on Days 2 and 4 of adulthood. Values are shown as arbitrary units. *elt-2* mRNA levels are significantly lower following *elt-2(RNAi)*. (* = Bonferroni-corrected p < .05, Student’s t-test). Error bars indicate SEM.
(PDF)

S7 Fig Direct binding of UNC-62 to the *elt-2* upstream region. Screenshot of modENCODE UNC-62:GFP ChIP-seq data (www.modencode.org) uploaded to the UCSC Genome Browser. The plot depicts UNC-62:GFP read densities from ChIP-seq upstream of *elt-2*. The top track represents a genomic scale bar. The second black track depicts the combined read density from two biological replicates. The third, blue track displays the *elt-2* gene model, with the exons as thick blue boxes. The UTR is represented as a thinner blue box. The bottom, black track shows significant binding peaks, relative to an input control, which were identified using PeakSeq (q-value≤10^−5^). Data were produced by modENCODE from worms at the Young Adult stage [54].
(PDF)

S8 Fig ELT-2:GFP levels decline during aging in *unc-62(RNAi)* animals. Bar graph shows quantitation of average GFP intensity from SD1949, which carries the modENCODE ELT-2 fosmid. Bar graph shows quantitation of average GFP intensity per animal from a population of 10–20 animals for each time point. Solid green bars indicate ELT-2:GFP expression in animals fed dsRNA against an empty vector control. Striped green bars indicate ELT-2:GFP expression in animals fed dsRNA against *unc*-62. RNAi was started at Day 1 of adulthood and images were taken at the indicated time. Error bars indicate SEM. *unc-62(RNAi)* causes ELT-2:GFP levels to be significantly higher at Day 4 of adulthood compared to control RNAi (p < .05, Student’s t-test). ELT-2:GFP expression at Day 8 is less than Day 1 for both control and *unc-62(RNAi)* animals.
(PDF)

S9 Fig Impact of *elt-7* RNAi on *elt-2* levels and lifespan. (A) Abundance of *elt-7* RNA, measured by qRT-PCR, in Day 2 adult animals, after 24 hours of feeding bacteria expressing dsRNA against *elt-7* or an empty vector control (L4440). *tbb-2* was used as a normalization control. Error bars indicate SEM from three biological replicates. The abundance of *elt-7* RNA was reduced by *elt-7(RNAi)* (p < .05, Student’s t-test). (B) Abundance of *elt-2* RNA, measured by qRT-PCR, in Day 2 adult animals, after 24 hours of feeding bacteria expressing dsRNA against *elt-7* or an empty vector control (L4440). *tbb-2* was used as a normalization control. Error bars indicate SEM from three biological replicates. (C) Lifespan of animals fed bacteria expressing dsRNA against *elt-7* or an empty vector control (L4440), beginning at Day 1 of adulthood. Lifespan assay was initiated with 100 animals per condition. Lifespan data are in S4 Table.
(PDF)

S10 Fig Enrichment of intestinal genes in ELT-2 ChIP-seq data. Venn Diagram depicts overlap between the 2484 low-complexity ELT-2 ChIP-seq targets and the 624 intestinally-enriched genes from Pauli et al., 2006.
(PDF)

S1 Table Low-complexity ELT-2 ChIP-seq targets. (XLSX)

S2 Table Age-regulated ELT-2 ChIP-seq targets. (XLSX)

S3 Table Single-cell ELT-2:GFP expression data. (XLSX)

S4 Table Lifespan data. (XLSX)

S5 Table List of genes differentially expressed between *elt-2(RNAi)* and control. (XLSX)

S6 Table Classification of *elt-2*-regulated genes. (XLSX)

S7 Table Slopes and p-values from linear regression analysis. (XLSX)

S8 Table Sources of tissue-specific gene lists. (XLSX)

S9 Table Long-lived mutants used for *elt-2* expression level analysis. (XLSX)

S10 Table Strain list. (XLSX)

S11 Table GEO accession numbers. (XLSX)
