# Supplementary figures and images for: Deactivation of the GATA Transcription Factor ELT-2 Is a Major Driver of Normal Aging in C. elegans
Source: PLoS Genet. 2016 Apr 12;12(4):e1005956. doi: 10.1371/journal.pgen.1005956 (PMC4829211; doi:10.1371/journal.pgen.1005956)

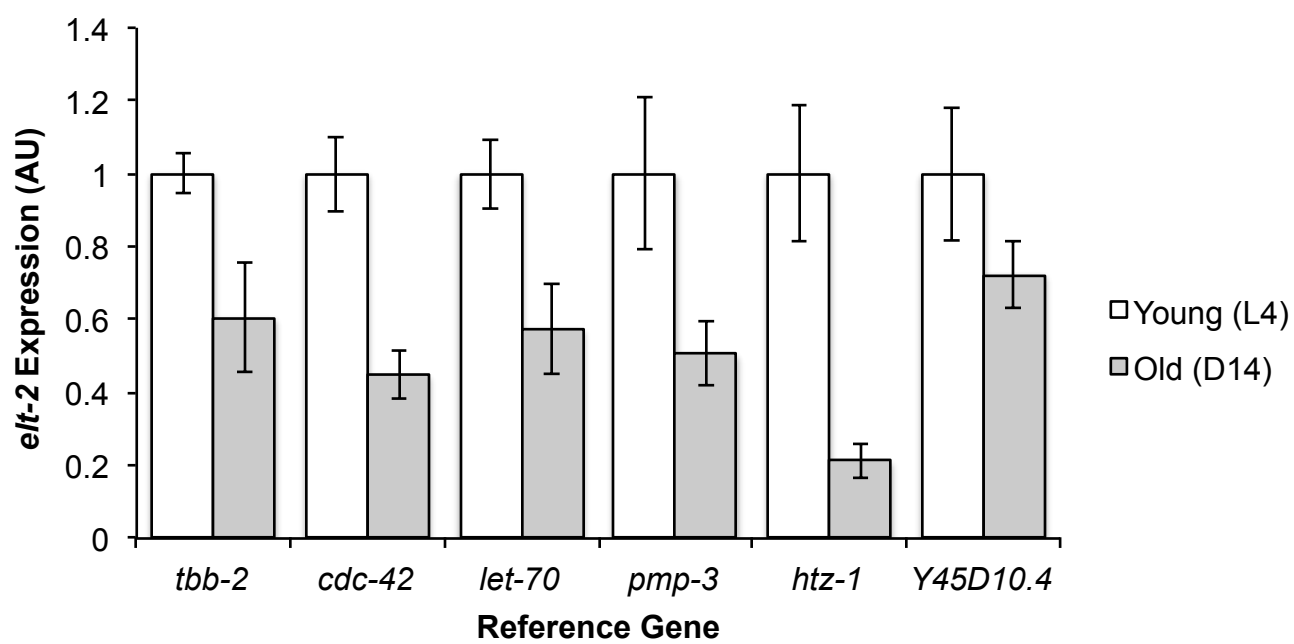

Supplement: S1 Fig — We measured levels of elt-2 mRNA by qRT-PCR in young and old using six different genes as controls. qRT-PCR was performed on mRNA extracted from three biological replicates (with three technical replicates per biological replicate) of young (L4) and old (Day 14) wild-type worms. elt-2 levels for young and old animals were measured using each reference gene as a control. The young timepoint was normalized to 1. Error bars indicate SEM. (PDF) [file pgen.1005956.s001.pdf]

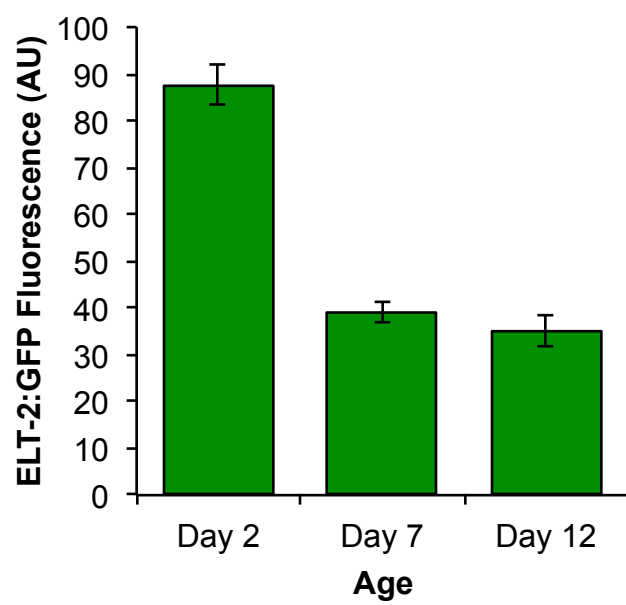

Supplement: S2 Fig — Bar graph shows quantitation of average ELT-2:GFP intensity per animal from SD1989. SD1989 contains the ELT-2:GFP fosmid constructed by modENCODE integrated at a low copy number and is homozygous for rde-1(ne300). 10–20 animals were used for each time point. GFP levels were measured by fluorescence microscopy and intensity levels were analyzed by ImageJ as described in Methods. Error bars indicate SEM. ELT-2:GFP levels are lower at Day 7 and Day 12 than at Day 1 (p < .05, Student’s t-test). (PDF) [file pgen.1005956.s002.pdf]

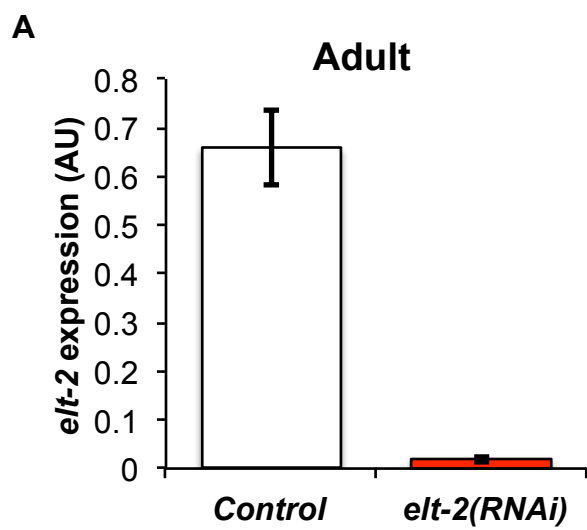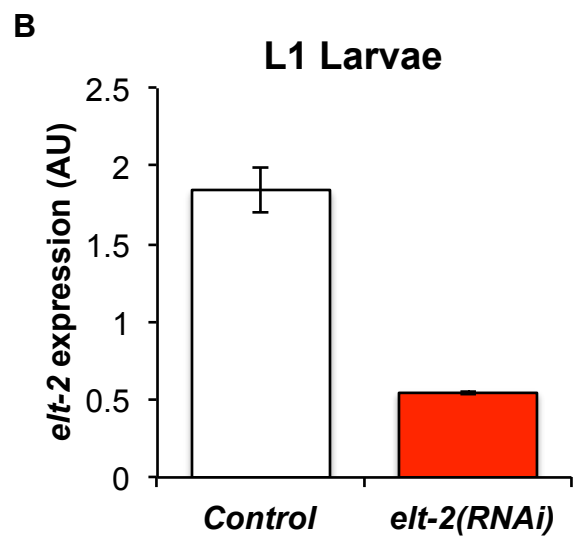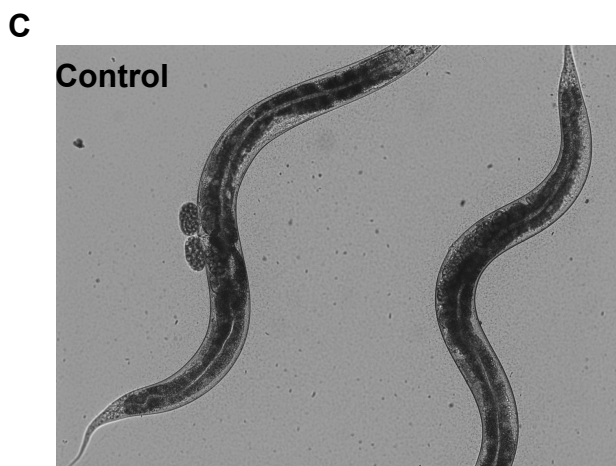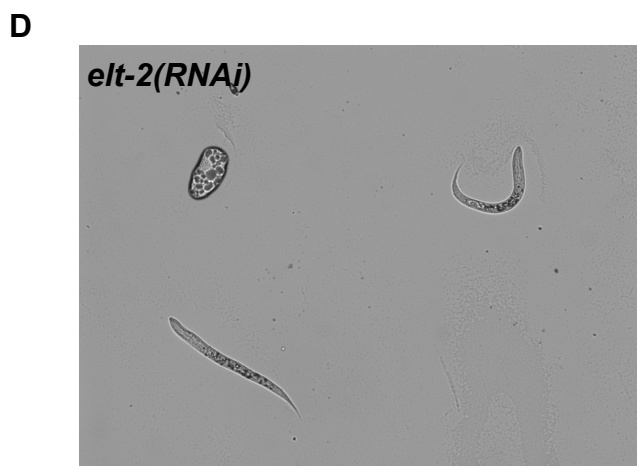

Supplement: S3 Fig — (A) Abundance of elt-2 RNA in adult animals fed dsRNA against elt-2 or an empty vector control starting at Day 1 of Adulthood, measured by qRT-PCR. tbb-2 was used as a reference. Error bars indicate SEM from three biological replicates. The abundance of elt-2 RNA is reduced by elt-2(RNAi) (p < .05, Student’s t-test). (B) Abundance of elt-2 RNA in wild-type controls and L1 larvae that are the progeny of adult wild-type hermaphrodites fed dsRNA against elt-2 or an empty control vector, measured by qRT-PCR. Error bars indicate SEM from three biological replicates. The abundance of elt-2 RNA is reduced by elt-2(RNAi) (p < .05, Student’s t-test). (C/D) Shown are progeny (3 days after hatching) from adult wild-type hermaphrodites that were fed dsRNA against elt-2 (C) or against an empty control vector (D). For elt-2(RNAi), approximately 75% of progeny showed larval-arrest. (PDF) [file pgen.1005956.s003.pdf]

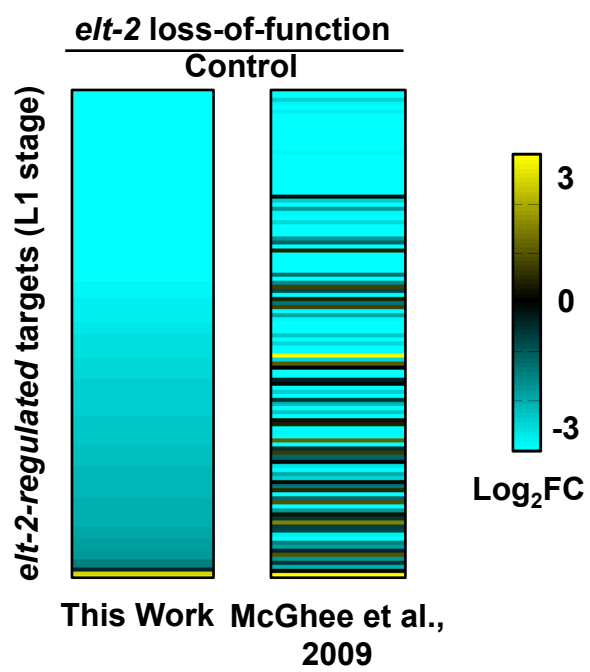

Supplement: S4 Fig — The two heatmaps show log2 expression changes between elt-2 loss-of-function and control animals for the elt-2-regulated genes at the L1 stage. The left heatmap shows expression changes between elt-2 RNAi and control-fed animals at the L1 stage, from RNA-seq data produced in this paper (average fold-change in from three biological repeats). The right heatmap shows expression changes between an elt-2 null mutant and wildtype L1 larvae, from SAGE data from McGhee et al., 2009 (fold-change from a single repeat). Shown is the concordance between the two data sets, defined as genes with expression changes of the same sign in both conditions. (PDF) [file pgen.1005956.s004.pdf]

**A****Development**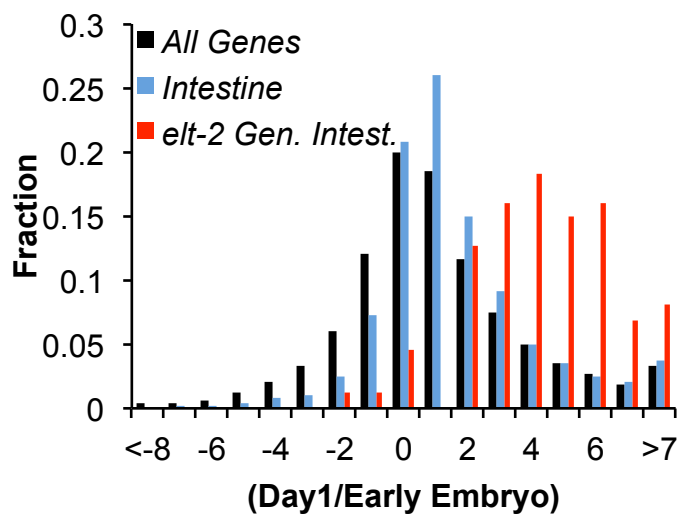**B****Aging**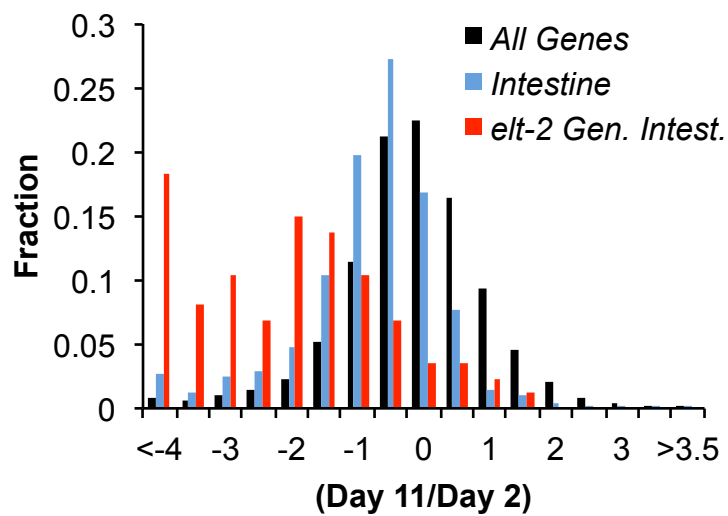

Supplement: S5 Fig — (A) Histogram of gene expression changes during development. Data are presented as the Log2 ratio of relative abundance at Day 1 of adulthood compared to early embryos, using RNA-seq data from modENCODE [28]. Three series are plotted: all genes (black), 1618 intestinally-expressed genes from Pauli et al., 2006 (cyan), and 88 elt-2 General Intestinal genes (red)[26]. The General Intestinal genes tend to be induced during development more than either intestinal-expressed genes or other genes in the genome (Kolmogorov-Smirnov p-value, General Intestinal vs. all genes: 1.7x10^-33^, General Intestinal vs. intestine-expressed genes: 1.8x10^-27^). (B) Histogram of gene expression changes during aging. Data are presented as the Log2 ratio of relative abundance at Day 11 of adulthood compared to Day 2 of adulthood, using DNA microarray data from Budovskaya et al., 2008. Three series are plotted: all genes (black), 1618 intestinally-expressed genes (from Pauli et al., 2006), and 75 General Intestinal genes (red) [26]. The levels of the General Intestinal genes tend to decline during aging more than either intestinal-expressed genes or other genes in the genome (Kolmogorov-Smirnov p-value, General Intestinal vs. all genes: 3.2x10^-29^, General Intestinal vs. intestine-expressed genes: 3x10^-17^). (PDF) [file pgen.1005956.s005.pdf]

**A**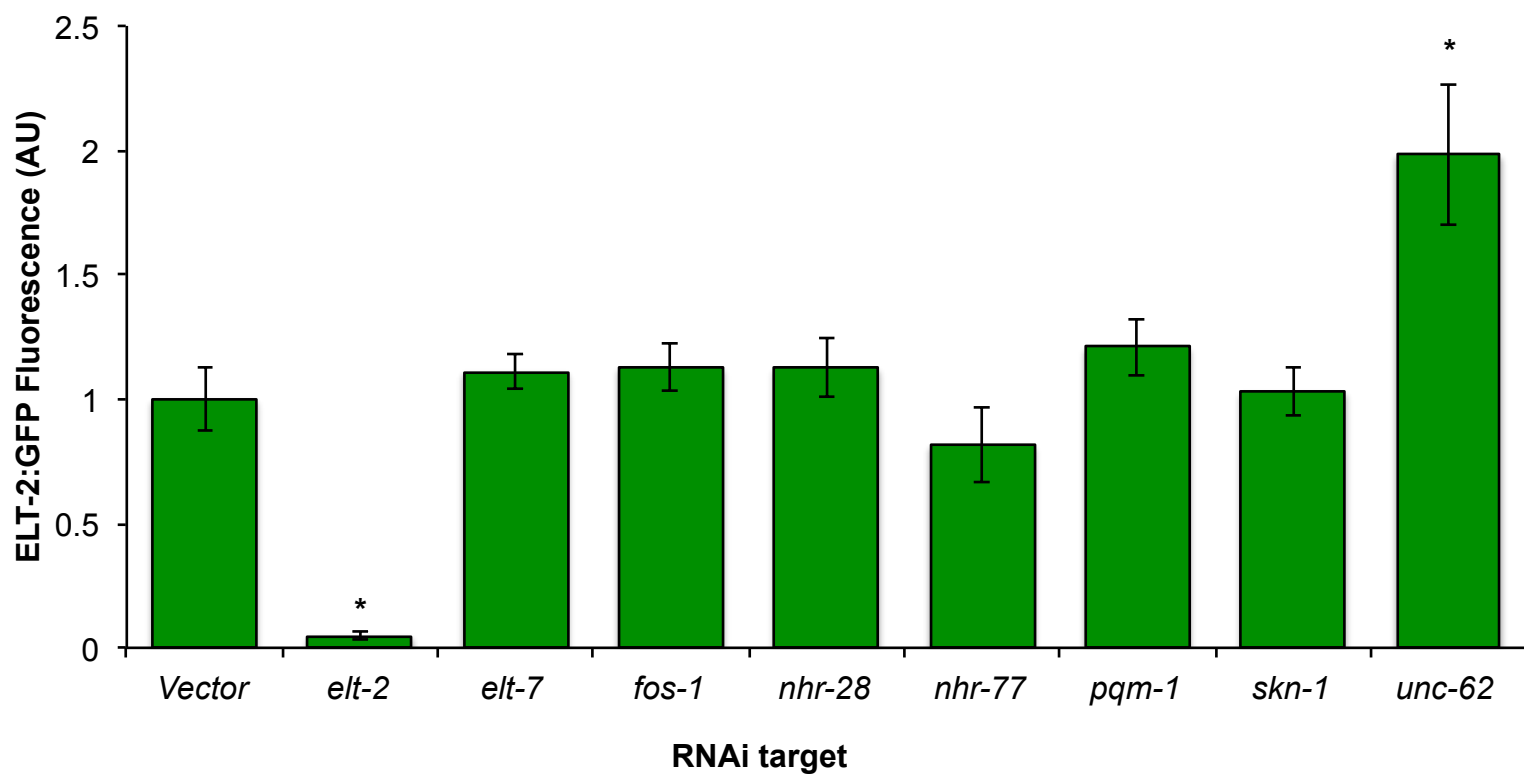**B**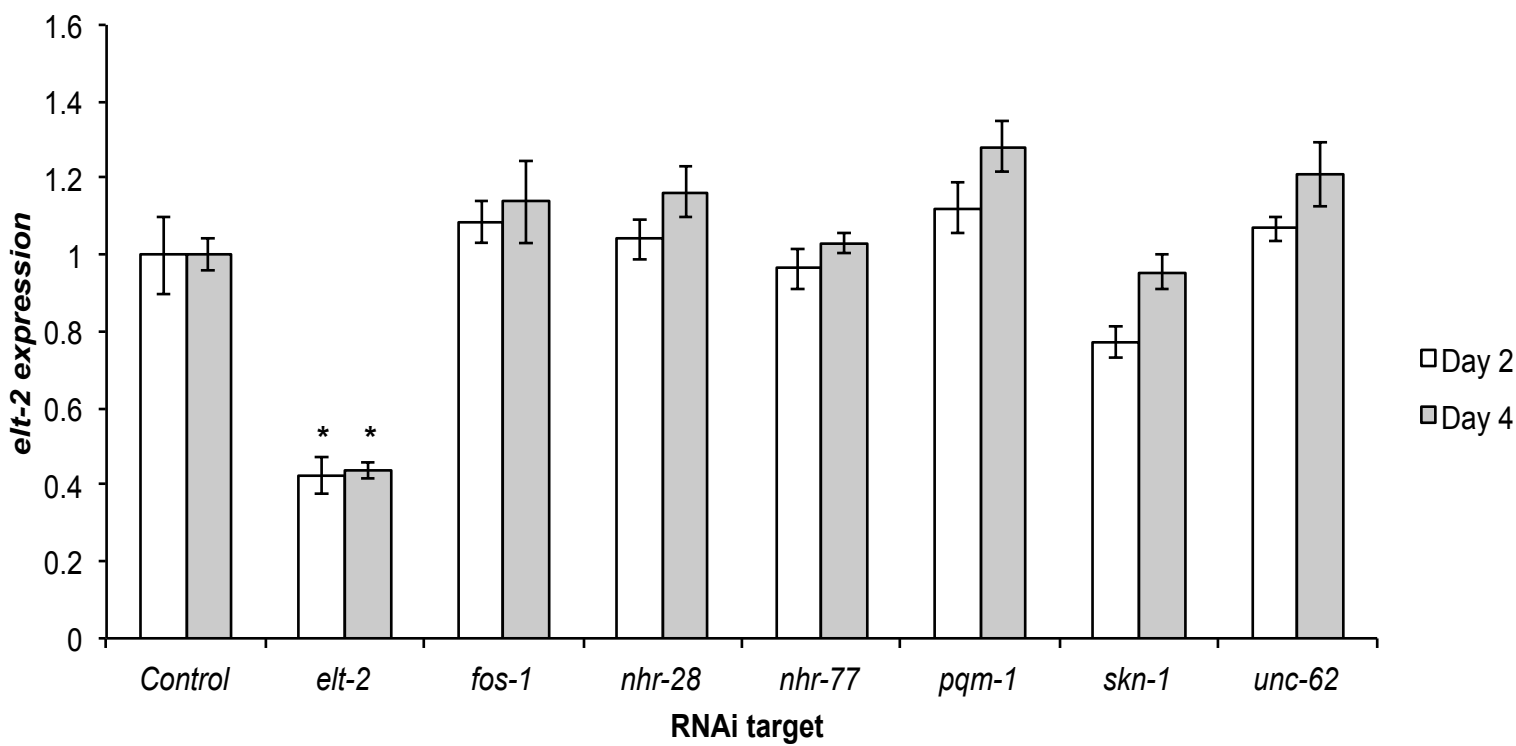

Supplement: S6 Fig — (A) Bar graph shows quantitation of average GFP intensity of SD1949, which carries the modENCODE ELT-2:GFP fosmid. Adult animals were fed dsRNA against the gene sequence corresponding to a given transcription factor at Day 1 of adulthood and GFP expression was measured at Day 2 of adulthood. Values are shown as arbitrary units indicating GFP levels per animal from a population of 10–20 animals. ELT-2:GFP levels are significantly lower following elt-2(RNAi) and significantly higher following unc-62(RNAi) (* = Bonferroni-corrected p < .05, Student’s t-test). Error bars indicate SEM. (B) Bar graph shows quantitation of average elt-2 mRNA relative to tubulin. Adult animals were fed dsRNA against the gene sequence corresponding to a given transcription factor at Day 1 of adulthood. Samples of 20–30 worms were collected on Days 2 and 4 of adulthood. Values are shown as arbitrary units. elt-2 mRNA levels are significantly lower following elt-2(RNAi). (* = Bonferroni-corrected p < .05, Student’s t-test). Error bars indicate SEM. (PDF) [file pgen.1005956.s006.pdf]

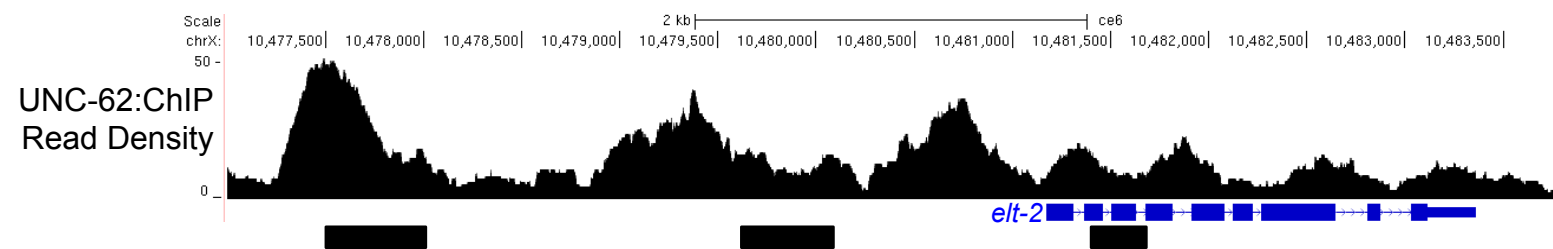

Supplement: S7 Fig — Screenshot of modENCODE UNC-62:GFP ChIP-seq data (www.modencode.org) uploaded to the UCSC Genome Browser. The plot depicts UNC-62:GFP read densities from ChIP-seq upstream of elt-2. The top track represents a genomic scale bar. The second black track depicts the combined read density from two biological replicates. The third, blue track displays the elt-2 gene model, with the exons as thick blue boxes. The UTR is represented as a thinner blue box. The bottom, black track shows significant binding peaks, relative to an input control, which were identified using PeakSeq (q-value≤10−5). Data were produced by modENCODE from worms at the Young Adult stage [54]. (PDF) [file pgen.1005956.s007.pdf]

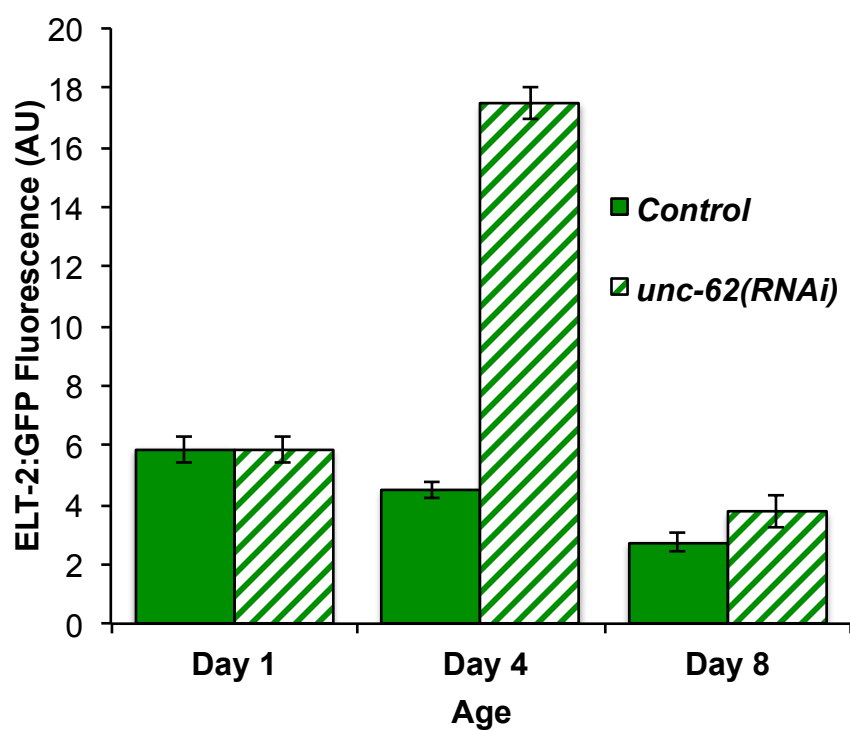

Supplement: S8 Fig — Bar graph shows quantitation of average GFP intensity from SD1949, which carries the modENCODE ELT-2 fosmid. Bar graph shows quantitation of average GFP intensity per animal from a population of 10–20 animals for each time point. Solid green bars indicate ELT-2:GFP expression in animals fed dsRNA against an empty vector control. Striped green bars indicate ELT-2:GFP expression in animals fed dsRNA against unc-62. RNAi was started at Day 1 of adulthood and images were taken at the indicated time. Error bars indicate SEM. unc-62(RNAi) causes ELT-2:GFP levels to be significantly higher at Day 4 of adulthood compared to control RNAi (p < .05, Student’s t-test). ELT-2:GFP expression at Day 8 is less than Day 1 for both control and unc-62(RNAi) animals. (PDF) [file pgen.1005956.s008.pdf]

**A**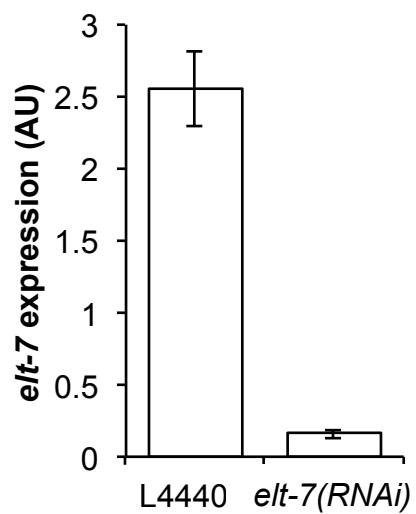**B**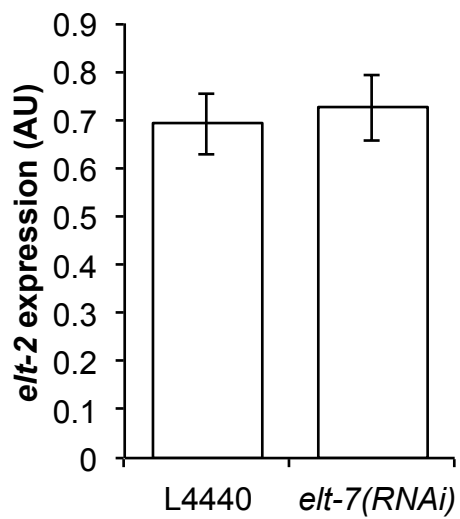**C**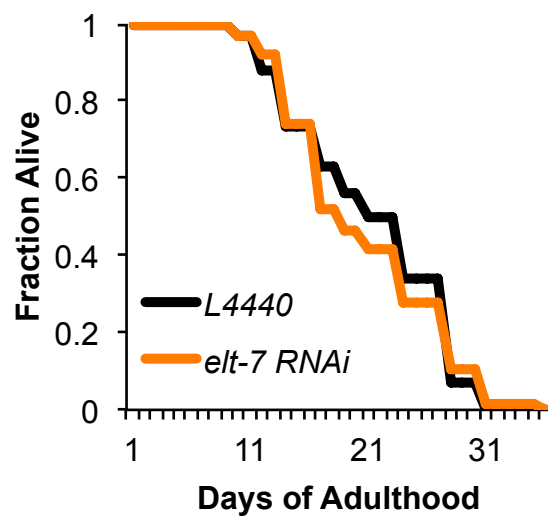

Supplement: S9 Fig — (A) Abundance of elt-7 RNA, measured by qRT-PCR, in Day 2 adult animals, after 24 hours of feeding bacteria expressing dsRNA against elt-7 or an empty vector control (L4440). tbb-2 was used as a normalization control. Error bars indicate SEM from three biological replicates. The abundance of elt-7 RNA was reduced by elt-7(RNAi) (p < .05, Student’s t-test). (B) Abundance of elt-2 RNA, measured by qRT-PCR, in Day 2 adult animals, after 24 hours of feeding bacteria expressing dsRNA against elt-7 or an empty vector control (L4440). tbb-2 was used as a normalization control. Error bars indicate SEM from three biological replicates. (C) Lifespan of animals fed bacteria expressing dsRNA against elt-7 or an empty vector control (L4440), beginning at Day 1 of adulthood. Lifespan assay was initiated with 100 animals per condition. Lifespan data are in S4 Table. (PDF) [file pgen.1005956.s009.pdf]

**ELT-2 ChIP Targets**

**Intestinally-Enriched Genes**

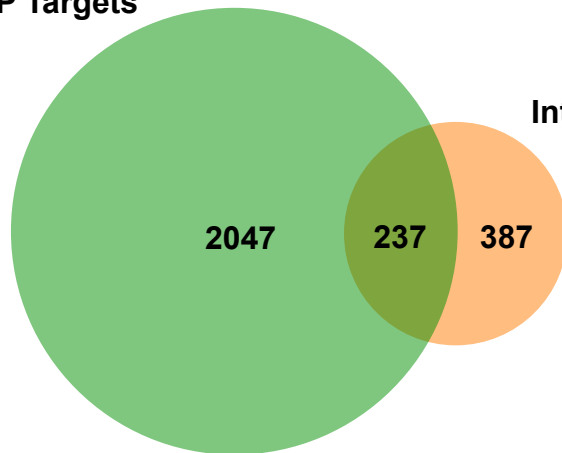

**3.1-fold enrichment**  
**Hypergeometric  $p = 1.8 \times 10^{-62}$**

Supplement: S10 Fig — Venn Diagram depicts overlap between the 2484 low-complexity ELT-2 ChIP-seq targets and the 624 intestinally-enriched genes from Pauli et al., 2006. (PDF) [file pgen.1005956.s010.pdf]
